# Who Gets the Guns? How Democratic Values and Security Threats Affect American Attitudes Toward Military Aid

**DOI:** 10.1177/00220027251388634

**Published:** 2025-10-28

**Authors:** Scott Williamson

**Affiliations:** 1 Department of Politics and International Relations and Magdalen College, University of Oxford, Oxford, UK

**Keywords:** foreign aid, terrorism, domestic politics, foreign policy, human rights, democracy

## Abstract

The United States gives substantial aid to the militaries of autocratic governments that abuse human rights. US officials claim this aid is necessary to manage security threats, but others argue the United States should prioritize aid for governments that reflect democratic values. How do these competing concerns shape Americans’ attitudes toward military aid? Through an experiment implemented on four surveys, I document a strong preference for aiding democracies that respect human rights, and this preference is robust to the presence of terrorism threats. However, internationalist Americans become especially less likely to prioritize democratic values when terrorist threats exist. Descriptive survey questions reinforce this pattern by showing how internationalists who support military aid the most are conflicted proponents of prioritizing democratic values in US foreign policy. The article extends research on attitudes toward foreign aid and illustrates an important limitation to the influence of democratic values on Americans’ foreign policy preferences.

## Introduction

In the months following Egypt’s coup in July 2013, Egyptian security forces massacred hundreds of peaceful protesters opposed to the country’s new military-led regime. The Obama administration responded to these events by suspending key portions of its military assistance to the Egyptian government. Within 2 years, however, the suspended aid had been restored, despite the Egyptian regime’s consolidation of authoritarianism and ongoing political repression. The administration justified this decision by pointing to an ISIS-affiliated insurgency that was gaining strength across the country (Ackerman 2015).

US security assistance for Egypt reflects a long-running pattern with US foreign policy, especially regarding aid for foreign militaries and security forces. The US government has spent billions on military assistance annually since the end of World War II (Sullivan et al. 2011), with yearly outlays ranging from $10 to $20 billion during the Cold War and then the War on Terror. This aid has frequently gone to the security forces of dictators who routinely violate human rights, and US officials have justified these aid relationships by emphasizing security interests, particularly related to the Soviet Union and later terrorism (Sandholtz 2016). Such relationships have persisted despite drawing negative public attention. Human rights organizations, members of congress, media, and the public at large have often criticized these policies, arguing that the United States should not send aid to governments that violate democratic values (e.g., Human Rights Watch 2023). In light of these debates over US military aid, how do these competing concerns shape the American public’s attitudes toward aid for foreign militaries? Do Americans tend to prioritize alignment with democratic values or management of security threats like terrorism in their preferences for military aid, and do they become less likely to emphasize democracy and human rights when salient security interests become more relevant to these aid relationships?

An established literature explores how public opinion toward foreign aid is shaped by various factors, including the recipient government’s alignment with democratic values or the presence of economic and security concerns. However, this research has focused predominantly on views of development and humanitarian assistance, or foreign aid generally, rather than on views of aid for militaries (e.g., Allendoerfer 2017; Bush and Zetterberg 2021; Dasandi et al. 2022; Heinrich et al. 2018; Heinrich and Kobayashi 2020; Powers et al. 2022; Stoll et al. 2023). Whether Americans prefer to send military aid to governments that are democratic and respect human rights, and whether the importance of these values is conditional on the existence of security threats, is an open question. On the one hand, a substantial amount of research finds that the foreign policy preferences of the American public are influenced by concern for democracy and human rights, including for issues involving war and conflict (e.g., Allendoerfer 2017; Bush and Zetterberg 2021; Doherty et al. 2020; Tomz and Weeks 2013, 2020). As a result, it is plausible that alignment with democratic values will outweigh security concerns in Americans’ attitudes toward military aid as well. Yet, other research on foreign aid indicates that many Americans are not averse to aiding unsavory regimes for instrumental reasons, including security interests (Heinrich and Kobayashi 2020). Given that military aid is tied more explicitly to national security policy, it is plausible that democratic values will be less relevant to attitudes toward this specific type of foreign assistance, especially when security concerns are salient. Thus, studying public opinion toward military aid can provide important insights into the scope conditions under which Americans prioritize democracy and human rights in their views of foreign aid as well as international affairs more broadly.

I also argue in this article that the influence of democratic values will be more conditional for some Americans than others. Specifically, I argue that Americans with an internationalist foreign policy orientation will be more likely than those with isolationist views to prioritize aiding democracies with good human rights records, but only when aid is not tied to salient security threats. When such threats are present, internationalists, more so than isolationists, will shift toward less emphasis on the recipient government’s alignment with democratic values. This conditionality should occur because most American internationalists favor some form of a US-led international order in which the US government takes the lead in both promoting democracy and managing threats to global security. These views create a contradiction in which the Americans who are most likely to care about using foreign aid to reinforce democracy and human rights in some circumstances are also the most likely to believe that military assistance for autocratic regimes can be necessary to address security challenges like terrorism. As a result, when security threats are relevant to military aid for a recipient country, internationalists should be more likely than isolationists to let go of their preference for aiding democratic governments that respect human rights, even if that preference is stronger in the absence of such threats.

The article provides evidence consistent with this argument through a series of experimental and descriptive survey results. The experiment was conducted on four separate surveys in 2016, 2017, 2019, and 2024. The descriptive analysis draws on questions from surveys administered in 2019, 2021, 2023, and 2024. Regarding the experiment, respondents were shown a hypothetical scenario in which the United States was said to be considering assistance for the military of a friendly foreign government. The scenario randomized the recipient country’s regime type and whether a threatening terrorist group was active in the country or not. The results reveal a strong preference for providing security assistance to a democracy that respects human rights over an autocracy that violates rights, and this effect is larger in magnitude than the effect of the threat treatment on all four surveys. Furthermore, the democracy and human rights effect is robust to the existence of the terrorist threat in the recipient country, weakening somewhat but remaining substantively meaningful. However, in three of the four surveys, this robustness masks conditionality based on respondents’ foreign policy orientation. Isolationist respondents did not demonstrate a noticeably weaker preference for assisting the democracy in response to the threat treatment. On the other hand, internationalist respondents reacted to the threat treatment by placing less emphasis on the recipient's alignment with democratic values. This pattern was present but much weaker in the fourth iteration of the experiment.

The descriptive results align with these findings, illustrating the importance of foreign policy orientation for shaping attitudes toward military aid and highlighting the tensions inherent in internationalists’ views. Across all four surveys in which the descriptive questions were asked, the results show that internationalists are substantially more likely than isolationists to believe both that US foreign policy should promote democracy and that the US government should be willing to work with the militaries of autocratic governments to fight terrorist groups. The internationalists who hold these two views simultaneously are the Americans who are most supportive of sending aid to foreign militaries. These patterns suggest that the constituency most likely to advocate for a US foreign policy that emphasizes democracy and human rights is also the most willing to send aid to autocracies in response to security threats like terrorism.

The findings have several implications for research on public opinion in international relations. First, they contribute to an important research agenda on the relevance of democracy and human rights to foreign policy preferences of the American public. Several influential studies have documented that Americans prefer US foreign policy options that reward and engage with countries that adhere to democratic values (Blackman 2018; Johns and Davies 2012; Rousseau 2005; Tomz and Weeks 2013, 2020). Whether it is preferences regarding whom to fight, which alliances to forge, or where to send foreign aid, research indicates a consistent desire to align with democracies and oppose autocracies in international affairs. On the one hand, my findings reinforce the robustness of this preference for democracy and human rights. Even for a security-oriented policy like military assistance, the recipient government’s alignment with these values appears to structure Americans’ preferences more strongly than the existence of a salient threat like terrorism. However, the findings also point to an important limit to the influence of these democratic values by revealing conditional effects of security threats based on foreign policy orientation. Especially since internationalists have historically been predominate in the US foreign policy elite (Chaudoin et al. 2010), these reactions suggest an important limit to how alignment with democratic values will influence Americans’ attitudes toward international affairs.

In developing these findings, the article also contributes to research on foreign policy orientation and its relevance to American public opinion. Scholars have established the continued importance of internationalist views to both elites and the public in the United States (Anderson and Garrison 2024), while also deepening our understanding of how foreign policy orientation interacts with domestic political ideologies to shape policy preferences (Chaudoin et al. 2010; Kertzer 2013; Prather 2024). The article extends this literature by showing that foreign policy orientation matters substantially more than partisanship for explaining attitudes toward military aid policies. The findings also contribute to research on foreign aid generally. Previous studies have shown that partisanship is less important for views of security assistance compared to other types of foreign aid (Milner and Tingley 2015), but relatively few studies in this literature have focused on who is more likely to support security assistance. Given the public controversy that often arises over military aid, as well as the negative repercussions for recipient countries that have been linked to US security assistance, it is important to understand who is more likely to support these aid relationships and why. In exploring how foreign policy orientation, democratic values, and security threats relate to support for military aid, the study illustrates how attitudes toward security assistance in the United States are both similar to and different from attitudes toward other types of aid.

The findings also speak to research that considers how threats affect support for democracy and democratic values more broadly. Scholars have previously established that security concerns can weaken the public’s commitment to key civil liberties at home (Davis and Silver 2003), particularly for out-groups (Carriere et al. 2022), but also that such effects tend to be modest (Godefroidt 2023). My findings show a similar pattern regarding support for structuring US foreign policy around engagement with foreign governments that reflect democratic values: respondents consistently place more emphasis on democracy and human rights than the terrorist threat in their support for sending military aid, and the strength of this preference for democratic values is relatively robust to the existence of the terrorist threat. At the same time, the conditionality by foreign policy orientation also illustrates the importance of identifying whose commitments to democratic values will be more sensitive to security threats and other contextual factors.

The article proceeds as follows. In the next section, I provide background information on US military aid. I then review literature on the relevance of democratic values versus more instrumental concerns to support for foreign aid, while developing my argument about how the connection between foreign policy orientation, security threats, and democracy shapes these aid attitudes. The following section describes the experimental design, after which I present the results of the experiment. I then discuss the descriptive findings that further link foreign policy orientation to support for military assistance. The article concludes with additional discussion of the findings’ implications for the role of democracy and human rights in US foreign policy.

## US Military Aid

The US government has several programs for providing security assistance to foreign militaries and security forces. These programs offer direct financing for purchases of military equipment and training programs for security personnel, as well as loans, leases, and sales of US defense services (Omelicheva et al. 2017). Large-scale provision of military aid began following World War II and quickly became a key foreign policy tool for the US government to counter Soviet influence during the Cold War (Sandholtz 2016). After annual spending dipped during the 1990s, it increased again following the September 11 attacks, with military aid becoming a cornerstone of US counterterrorism policy in countries experiencing insurgencies and terrorism (Sullivan 2023). In explaining the rationale for military aid, the US Department of Defense primarily emphasizes national security interests. For instance, foreign military financing programs are described as a tool to ensure that “coalition partners and friendly foreign governments are equipped and trained to work toward common security goals and share burdens in joint missions,” while training of foreign officers is a way to promote “regional stability and defense capabilities through professional military training and education” (Office of Security Assistance 2023). Some research indicates these programs can be effective at achieving strategic aims, for instance by increasing US soft power among foreign military elites and securing closer alignment with US interests in international affairs (Martinez Machain 2021). However, in addition to such interests, the Department of Defense also argues that these programs contribute to the proliferation of US values by exposing officers to democratic governance and training them to respect human rights standards (Office of Security Assistance 2023). Researchers have assessed this claim and found mixed results, with some studies suggesting that US military training tends to inculcate more democratic officers (e.g. Ruby and Gibler 2010), but several others finding that US training increases coup risks and imparts norms that can undermine democratic values and human rights practices (e.g. Grewal 2022; Joyce 2022; Savage and Caverley 2017).

In fact, despite legal restrictions on providing foreign aid to governments that violate human rights, US security assistance frequently flows to these governments anyways (Sandholtz 2016), and this aid represents one of the most direct ways in which the United States aligns itself with autocrats and contributes to rights violations abroad. Military assistance during the Cold War frequently supported security units and officers most responsible for torture, disappearances, and mass killings in their countries (Higgins 1998; McCoy 2005; Rabe 2012). In recent decades, Egypt, Jordan, Afghanistan, Iraq, and Israel have all been top recipients of US foreign assistance generally and military aid specifically, despite consistently violating democratic standards and human rights. Research indicates that US assistance for militaries and security forces is associated with increased human rights violations by the recipient governments (Dube and Naidu 2015; Omelicheva et al. 2017; Sandholtz 2016), especially when “lethal” aid is provided (Sullivan 2023). Given that robust security apparatuses contribute to the longevity of authoritarian regimes (Albertus and Menaldo 2012; Bellin 2004), military assistance may be particularly useful for helping authoritarian governments to repress opposition and maintain power.

Yemen provides an example of US security assistance contributing to human rights violations while propping up a friendly autocrat during the War on Terror. US-funded and trained counterterrorism units in the country – commanded by family members of the country’s dictator, Ali Abdullah Saleh – played a key role in protecting the regime from internal opponents. In the process, these units committed significant rights abuses, while also ignoring their ostensible mission to combat Al-Qaeda in the Arabian Peninsula (Scahill 2012).

Perhaps because of its clear relevance to human rights abuses, military assistance is frequently contested by human rights advocates in the United States. Organizations like Human Rights Watch often advocate for reducing military aid to abusive autocrats. Returning to the Yemen example, when Saleh’s forces violently repressed protesters during the Arab Spring, Human Rights Watch (2011) highlighted the $300 million in assistance that had been provided to the regime in the previous 5 years and called on the US government to cut off this support. Such advocacy also occurs in Congress, where some US Senators and Representatives consistently draw attention to military aid that flows to human rights violators. US assistance for the Saudi and Emirati war in Yemen is one case where influential members of Congress contested this support (Gambino Lauren and Borger 2019). These efforts often receive substantial press coverage and can draw the attention of the public. This advocacy and public attention should increase the likelihood that public opinion can influence policy in these cases (Heinrich et al. 2018; Nielsen 2013). Given the importance of military assistance to US foreign policy, the attention it receives from human rights advocates and the public, and its often-negative impact in recipient countries, it is worthwhile to study the considerations that influence Americans’ attitudes toward this type of aid.

## Democratic Values, Threats, and Preferences for Military Aid

While it was long assumed that the American public had relatively weak preferences regarding foreign policy, researchers have increasingly shown that citizens’ attitudes in this domain are coherent and can be influential in the domestic politics surrounding international affairs (Kertzer and Zeitzoff 2017; Milner and Tingley 2013; Powers and Renshon 2021; Shapiro and Page 1988). One such preference that is apparent in public opinion across numerous spheres of US foreign policy is to engage with states that reflect democratic values, including elected governments and respect for core human rights and political freedoms. For example, research indicates that Americans are much less likely to support going to war with democracies or countries that adhere to human rights standards (Johns and Davies 2012; Tomz and Weeks 2013, 2020). Likewise, Americans prefer to send foreign aid to democratic countries with good human rights records (Allendoerfer 2017; Blackman 2018; Doherty et al. 2020; Heinrich et al. 2018), and rhetoric about values of democracy and human rights can also increase the public’s support for foreign alliances or the International Criminal Court (Chu et al. 2021; Zvobgo 2019).

This preference for working with states that uphold democratic values can reflect both moral and strategic concerns (Brancati 2014). Many Americans hold a normative commitment to democracy; for instance, conflict with democratic governments is seen as less moral than conflict with autocracies (Tomz and Weeks 2013). Others also perceive the spread of democracy as beneficial to US power and security because it promotes a more stable and friendlier international system (Wollack 2008). The combination of these factors likely explains why democracy and human rights are relevant for many Americans in how they think about foreign policy issues generally. These moral and instrumental considerations are also both relevant to military aid. For those concerned with the normative consequences of security assistance, this aid can actively reinforce authoritarianism and increase the likelihood of serious human rights violations. Instrumentally, military aid for democracies plausibly strengthens military cooperation with governments that are more likely to maintain friendly relations with the United States and to promote other US security interests. Combined, these factors suggest that Americans’ attitudes toward military aid will reflect a preference to assist countries that adhere to democratic values.

However, despite the apparent importance of democracy and human rights to Americans’ foreign policy preferences, in practice US foreign policy has frequently supported foreign governments that are abusive and autocratic, with these relationships typically linked to key US security objectives (Hook 1998). As discussed in the previous section, this support has often come in the form of military assistance, though autocrats have received substantial development aid as well. Across a number of contexts, perceived threats can reduce the public’s commitment to democratic values (Carriere et al. 2022), and it is plausible that the American public has fewer qualms about aligning their country with abusive, autocratic governments in contexts where security interests are seen to be prevalent. In this vein, more recent work has explored how more direct, instrumental concerns about economic and security interests influence Americans’ aid preferences. For instance, Heinrich et al. (2018) and Allendoerfer (2017) use survey experiments to explore whether Americans are more likely to support aid for abusive governments if their countries provide the United States with security and economic benefits. Likewise, Doherty et al. (2020) rely on a conjoint experiment to study how a country’s level of political freedom shapes attitudes toward aid relative to more instrumental attributes such as the amount of US exports to the country. These studies indicate that factors such as security benefits can increase support for aiding autocratic or abusive governments. However, they also tend to show that the recipient’s alignment with democratic values still has a larger impact on Americans’ preferences about which countries should receive assistance.

To date, researchers have focused on views of development and humanitarian aid, or foreign aid generally, when studying public opinion in donor countries. Yet, military aid may be characterized by different patterns of support, since it is explicitly focused on a security-oriented sector. It is possible that Americans would place more emphasis on explicit security interests and would be less concerned about alignment with democratic values in their attitudes toward military aid, since they expect this type of assistance to address national security policy. As such, military aid should provide a hard test of the extent to which Americans care that foreign aid be given to recipient governments that align with democratic values.

It is also useful to consider whether certain Americans will weigh alignment with democratic values and the presence of direct security threats differently when thinking about which countries should receive military aid. Here, I argue that foreign policy orientation affects which Americans support military aid generally and which Americans are more likely to shift more toward acceptance of aiding autocracies in contexts where threats are relevant.

Broadly speaking, foreign policy orientation refers to whether Americans are internationalists who want the United States to take an active role in world affairs, or isolationists who want the United States to look inwards to focus on domestic policy. Scholars have recognized the importance of the internationalist-isolationist divide in American foreign policy for some time, and recent work indicates that it continues to be a relevant and relatively stable determinant of foreign policy views, even across ideological and partisan lines (Chaudoin et al. 2010; Kertzer 2013; Prather 2024). For instance, an internationalist orientation is strongly associated with higher support for development aid among conservative and liberal Americans alike (Prather 2024). In the decades following World War II, a majority of Americans expressed an internationalist orientation (Chaudoin et al. 2010), and nearly all American foreign policy elites were internationalist during this period (Page and Barabas 2000). This consensus remained relatively robust at least through the War on Terror (Busby and Monten 2008), and though it may have frayed somewhat under the first Trump presidencies (Dodson and Brooks 2022), it continues to be fairly resilient in terms of public opinion (Anderson and Garrison 2024).^1^

Not all internationalists view foreign policy the same way. There are ideological disagreements between those who prefer a more cooperative mode of international engagement and those who prefer a more militant approach (e.g., Chanley 1999; Holsti and Rosenau 1990; Kertzer et al. 2014). Nonetheless, despite disagreements over methods, most internationalists share a broad commitment to US leadership in the international system that involves working with allies to manage key global security challenges and promoting US values such as democracy, human rights, and open markets (Anderson and Garrison 2024; Ikenberry 2009; Maggiotto and Wittkopf, 1981; Wittkopf 1986).

In practice, these goals often conflict, insofar as the pursuit of international security frequently entails partnering with and supporting countries that do not align with US values and may actively work against them. This potential for conflict between internationalists’ interest in promoting both global security and democratic values should have implications for how internationalists think about military aid. As a matter of general policy, internationalists should be substantially more likely than isolationists to support providing funds and training for foreign militaries aligned with the United States. Because most internationalists want US foreign policy to reflect and promote democratic values, they should also be more likely than isolationists to prioritize military aid for democracies with good human rights records over autocracies that routinely violate rights.

However, since internationalists likewise want the United States to be active in managing global security, they should be more likely to believe the US government should involve itself in foreign conflicts that could become threats to the US-led international order, US allies, and the United States itself. Furthermore, military aid offers a tool that should appeal to both cooperative and militant internationalists: it is a cooperative policy built on US military power that can, at least in theory, bolster the ability of allied forces to fight insurgencies, engage in counterterrorism operations, address piracy, and deter hostile states, among other contributions to global and US security. And because internationalists care more than isolationists about addressing these global security threats, they should become more likely to perceive military aid for autocrats as justifiable when such threats are present. As a result, the effect of democracy and human rights on internationalists’ attitudes toward military aid should be more sensitive than those of isolationists to security concerns, with internationalists more likely to prioritize aid for democracies in the absence of salient threats, but also more likely to shift toward supporting military assistance for autocracies when challenges to global security become relevant.

I am therefore interested in answering three questions. First, do Americans place more emphasis on a recipient’s alignment with democratic values or the presence of explicit security threats when considering whether foreign countries should receive military aid? Second, does the presence of security threats weaken the extent to which Americans care about aiding governments that align with these values? And third, does this conditionality vary by foreign policy orientation, such that internationalists are particularly likely to deprioritize democracy and human rights when threats became relevant to military aid relationships? The following sections explain the survey data and experimental design used to evaluate these questions.

## Research Design

To test how alignment with democratic values and the presence of explicit security threats influence Americans’ preferences regarding military assistance, I combine analysis of experimental and descriptive survey questions. The experiment was embedded on four surveys implemented online in the United States. The first two surveys were conducted by YouGov with nationally representative samples of 1,000 American adults. The surveys were fielded in December 2016 and March 2017. The third survey was implemented on Qualtrics in April 2019 with a sample of 1,299 Americans recruited by Lucid. The fourth survey was run on Qualtrics in December 2024 with a sample of 3,958 Americans recruited by Bilendi. Replicating the experiment with different samples at different time periods and across three presidential administrations should strengthen confidence in the robustness and generalizability of the findings. The 2019 survey also included a series of descriptive questions about preferences toward military aid, democracy promotion, and combating terrorism that were intended to shed further light on the patterns identified by the experiments. These questions were then replicated on subsequent Qualtrics surveys with Lucid-recruited samples in 2021 and 2023, as well as the Bilendi survey in 2024. Table 1 provides a summary of the different surveys.

**Table 1.** Summary of surveys

	YouGov 2016	YouGov 2017	Lucid 2019	Lucid 2021	Lucid 2023	Bilendi 2024
Experiment	*✓*	*✓*	*✓*			*✓*
Descriptive			*✓*	*✓*	*✓*	*✓*
Sample size	1,000	1,000	1,299	1,983	1,532	3,958

The experiments presented respondents with a prompt about a situation in which the US government is considering providing funding and training to the military of a foreign government. Two primary manipulations were embedded in the prompt. For the first treatment, respondents were told that the country’s government was either a democracy with a reputation for respecting human rights or an autocracy with a reputation for violating human rights. Regime type and human rights can be treated as distinct, but this study combines them for several reasons. Conceptually, the two are closely linked. Even relatively minimalist definitions of democracy incorporate protections for core political freedoms that facilitate competitive elections (e.g., Lührmann et al. 2018), so autocracies will, by definition, have reputations for violating important rights. Democracies vary in the strength of their human rights records, but empirically they perform better than autocracies at providing rights to their citizens (Møller and Skaaning 2013). This approach also reflects popular understanding of democracy, as Americans and others view political rights as a core component of democratic governance (Chu et al. 2024). In addition, several of the countries that have attracted public controversy for receiving substantial US military aid are autocracies that have engaged in particularly severe violations of human rights.^2^ As a result, the combined treatment should be conceptually clearer for respondents and is more in line with how military aid is discussed in American political debates. This approach also reflects design choices by other studies that examine how democratic values broadly relate to foreign policy preferences (Chu et al. 2021). For simplicity, I refer to this condition as the “democratic values” treatment, by which I mean that the potential aid recipient aligns with democratic values.^3^ However, in the 2024 Bilendi survey, I also disaggregate democracy and human rights into different treatments to assess if the results change. The effects are similar for the individual regime type and human rights treatments, so I report disaggregated treatment effects in Supplemental Information-4.7 and the combined treatment effect in the main text.^4^ In the discussion section, I also briefly consider the implications of these results for attitudes toward aid for democracies that violate rights.

The second manipulation involved the level of threat. Respondents were told that there was no direct security threat to the United States emanating from the country, or that a terrorist group that had killed Americans was active in the country and being fought by the government’s military forces. I chose to mention a terrorist group as the security threat because most of the security assistance distributed to repressive regimes since 2001 has been justified by references to combating terrorism, and US aid flows are particularly responsive to the presence of terrorist groups that have directly threatened US interests (Boutton and Carter 2014). This treatment is also especially relevant because research from other contexts has shown that some Americans lessen their support for civil liberties and other democratic principles when confronted with terrorist threats (Davis and Silver 2003; Hetherington and Suhay 2011; Huddy et al. 2005). The text of the prompt was as follows:

As part of its foreign policy, the United States often provides financial and technical support to the militaries of friendly foreign governments. This support can contribute to US foreign policy objectives by helping to maintain close relationships with these governments. However, the US government has limited resources and must choose carefully whether a country should receive this support. Consider a common situation in which US officials are debating whether to fund and train the military of a country in [Africa/the Middle East/Asia]. Some details about the country and its government are provided below. Please read these details carefully and then answer the questions.

Following the prompt, respondents read four details about the country. The first two details—reliability and military—were kept constant. The second two details—human rights and US security—constituted the experimental manipulations of interest. The order of these latter two details was randomized to avoid order effects.^5^

• **Reliability:** The country’s government has been a reliable partner of the United States.
• **Military:** The country has a large but poorly trained military that would benefit from US assistance.
• **Human Rights:** The country’s government is democratic and has a reputation for respecting human rights/The country’s government is authoritarian and has a reputation for violating human rights.
• **US Security:** There are no direct threats to US security in this country/The country’s military is currently fighting a terrorist group that has attacked the United States and killed dozens of American citizens.

The body of the prompt, along with the reliability and military details, were designed to ensure that respondents would perceive positive reasons to provide the aid, regardless of the human rights record or the presence of a security threat. Security assistance has often been provided to loyal partners of the United States even in the absence of direct threats emanating from the country, so this profile also reflects a plausible representation of an aid relationship.

For the outcome, I am interested in whether respondents would support providing military assistance to the government in question. Thus, immediately after the prompt, respondents were asked the following: “Based on the information you just read, do you agree or disagree that the US government should fund and train this country’s military?” Answer choices were on a seven-point scale ranging from strongly agree to strongly disagree. Approximately 25 percent of respondents were opposed to assistance, 25 percent were neutral, and 50 percent were supportive. To simplify the analysis, answers are converted to a binary variable, with explicit supporters coded as 1 and neutrals and opposed coded as 0. Results are the same when using the ordinal variable, as shown in Supplemental Information-4. The treatment groups were balanced across several demographic and political covariates in the surveys.

The second survey included two minor modifications. First, to control for respondents’ perceptions of cost, the prompt randomly specified the amount of the aid: $10 million, $100 million, or $1 billion. Second, the threat treatment included three conditions rather than two: no security threat, a terrorist organization that had killed dozens of American citizens, and a terrorist organization that had killed hundreds of American citizens. Because these manipulations did not materially affect the outcomes, the third and fourth surveys reverted to the design of the first, and I combined the two threat conditions into one threat treatment when analyzing the second survey.

The region of the country was randomized in the first, second, and fourth surveys to provide some control over the countries inferred by the respondents.^6^ The terrorist group was not specified in the security condition, but it seems likely that most respondents would impute either Al-Qaeda or the Islamic State. Both of these organizations have attacked the United States and killed Americans, and both have had active affiliates in Africa, the Middle East, and Asia. The US government also provides military aid to governments in all three regions. As a result, the prompt should have been plausible even for well-informed respondents.

To identify internationalists, I asked respondents if they agree or disagree that “the United States should take a more active role in world affairs.” I define internationalists as those who affirmatively agree with this statement, and isolationists as those who disagree. I also show in Supplemental Information-4.6 that results are consistent with an additional measure that was included on the third survey, which asked respondents to choose between the United States taking an active role in world affairs or staying out of world affairs.

The survey experiment was not pre-registered. Instead, I rely on replication to strengthen confidence in the findings. For the sake of transparency, it is important to note that the argument about foreign policy orientation was derived from exploratory analysis of the first survey, after which I used the subsequent three replications and the descriptive analysis to develop and test the argument further.

For the descriptive component of the analysis, I asked respondents three questions: whether they support training and funding the militaries of friendly foreign governments, whether they believe democracy promotion should be a priority of US foreign policy, and whether they believe the US government should actively work to fight terrorism abroad by partnering with both democratic and authoritarian governments. I then analyze the extent to which internationalist Americans are more likely to hold all three views, reflecting the argument that they will have strong but fickle preferences for US foreign policy to align with democracy and human rights.

## Results

I first analyze the extent to which support for military aid is influenced by the recipient government’s alignment with democratic values or conflict with an anti-American terrorist group, comparing the magnitude of the effects for these two treatments. Next, I analyze whether the presence of the terrorist threat weakens Americans’ preferences for aiding the government that aligns with democratic values, and I assess whether this weakening is particularly likely to occur for internationalists. For the experiment, I analyze data combined across the four surveys as well as results for each survey. In the main text, I report results from simple models in which the binary measure of aid support is regressed on the democratic values and threat treatments, and I report results with control variables and other robustness checks in Supplemental Information-4.^7^ I then move to a discussion of the descriptive analysis relating foreign policy orientation to support for military aid.

### Main Effects of Democratic Values and Threat Treatments

**Figure 1.**
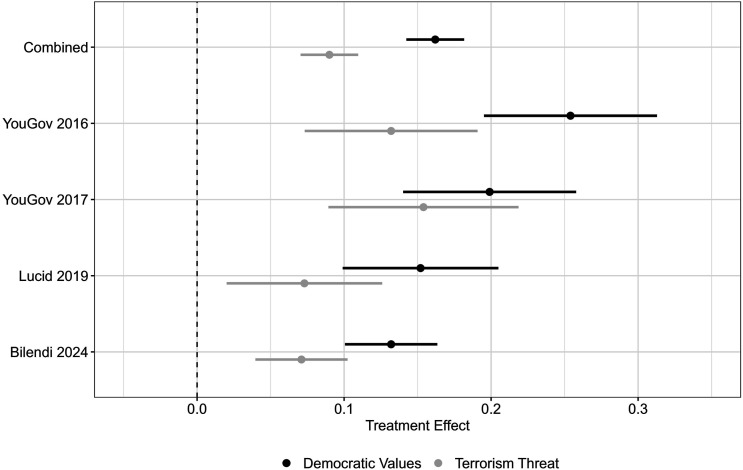
The values treatment increases aid support more than the threat treatment. *Note.* 95% C.I.

Figure 1 reports the main effects of the democratic values and threat treatments with the combined data and across the four surveys. As would be expected, both conditions increase support for providing military assistance to the potential recipient. Compared to the autocracy with the bad human rights record, the democracy with the good human rights record increases support for aid by approximately 16 percentage points in the combined data (*p* < .001), 25 percentage points in the first survey (*p* < .001), 20 percentage points in the second survey (*p* < .001), 15 percentage points in the third survey (*p* < .001), and 13 percentage points in the fourth survey (*p* < .001). Relative to the condition where no US security interests are present in the recipient country, the terrorism threat treatment increases support for military assistance by approximately 9 percentage points in the combined data (*p* < .001), 13 percentage points in the first survey (*p* < .001), 15 percentage points in the second survey (*p* < .001), 7 percentage points in the third survey (*p* < .01), and 7 percentage points in the fourth survey (*p* < .001).

Noticeably, the democratic values treatment effects are larger than those associated with the terrorism threat in all four surveys. These differences are statistically significant in the first, third, and fourth surveys, and they are substantively large as well, with the democratic values effect approximately twice as large as the threat effect in the first, third, and fourth surveys and approximately 30 percent larger in the second survey. These magnitudes suggest that the governance record of the recipient country outweighs concerns over terrorism threats – a security issue that has been highly salient in US politics – in shaping Americans’ support for military aid relationships.

### Effects of Democratic Values Conditional on Security Threat

**Figure 2.**
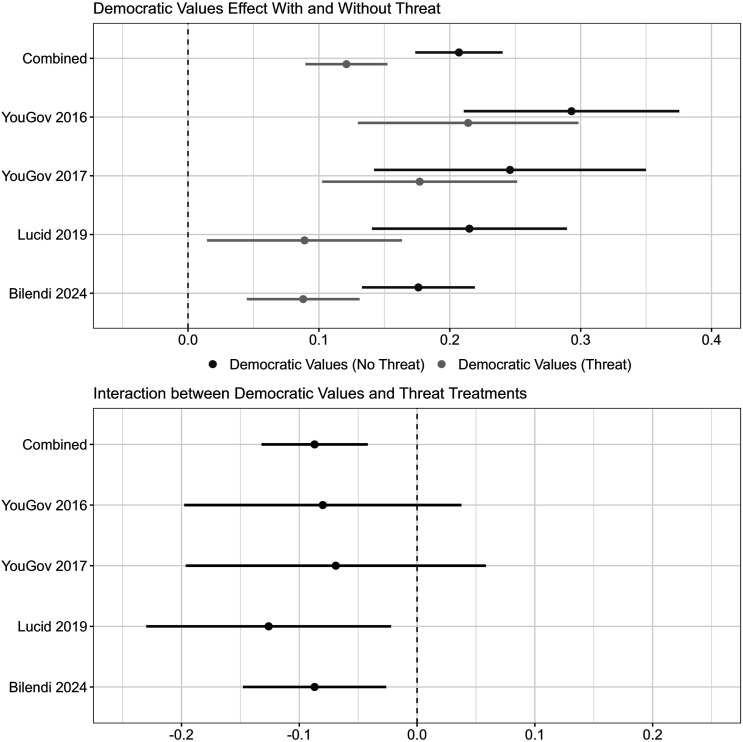
The democratic values effect is robust to the terrorism threat. *Note.* The democratic values treatment is weaker when the security threat is present, but it remains substantively meaningful in all four surveys. 95% C.I.

Does the existence of a hostile terrorist group reduce the strength of the relative preference for aiding the democracy with the reputation for respecting human rights? To assess the extent to which terrorism threats undermine the weight placed on democratic values, I analyze whether the terrorism threat condition reduces the magnitude of the democratic values treatment effect relative to when no direct security interests are present. Figure 2 displays these results. The top plot shows the democratic values treatment effects among respondents in the no threat condition and respondents in the threat condition for the combined data and each of the four surveys. The bottom plot shows the magnitude of the difference by displaying the interaction between the democratic values and threat treatments.

The results provide some support for the idea that terrorism threats weaken Americans’ preference for aiding democratic states with good human rights records, but they also indicate that the influence of democracy and human rights on attitudes remains strong even in the shadow of terrorism concerns. In all four surveys, the democratic values treatment effect becomes smaller when the country receiving the military aid is said to be fighting an anti-American terrorist group. In the combined data, the democratic values effect declines from 21 percentage points in the absence of direct threats to 12 percentage points (*p* < .001) when the terrorist group is present. For the first survey, the effect falls from 29 to 21 percentage points (*p* = .19); for the second survey, it is reduced from 25 to 18 percentage points (*p* = .29); for the third survey, it declines from 22 to 9 percentage points (*p* < .05); and for the fourth survey, it moves from 18 to 9 percentage points (*p* < .01). On the one hand, these reductions of the democratic values effect in the threat condition suggest that salient terrorism concerns can weaken the strength of the public’s preference for aiding democracies with good human rights records over autocracies that violate rights. On the other hand, the democratic values effect remains substantively meaningful even when the threat is present.

In other words, the American public appears to maintain a meaningful preference for aiding governments that align with democratic values regardless of whether terrorism concerns are salient or not, though threats may weaken this preference to some extent. This point is reinforced further by considering the percent of respondents in these conditions who supported the provision of military aid. In the combined data and in all four surveys, support is higher for aiding the military of a country where no terrorism threat is present and the government reflects democratic values than it is for aiding the military of a country where the anti-American terrorist group is active and the government is authoritarian and violates human rights.^8^

### Internationalism and the Democratic Values Effect

**Figure 3.**
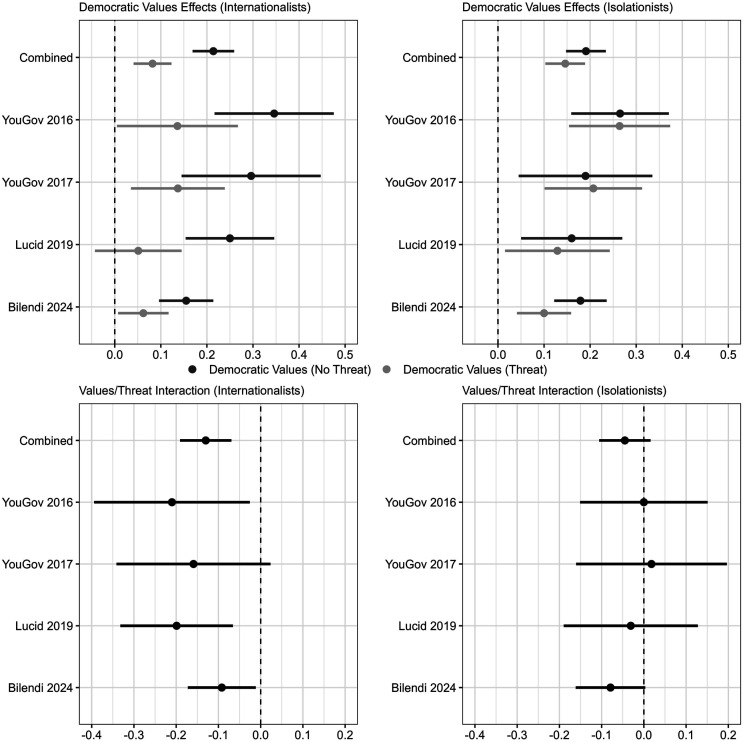
The threat treatment weakens the democratic values effect for internationalists. *Note.* The top plots show the democratic values effects among internationalists and isolationists when the terrorism threat is present or not. The bottom plots show the differences between these democratic values effects. Among internationalists, the democratic values treatment weakens substantially in the presence of the security threat in three of the four surveys and in the combined data. This same pattern does not occur among isolationists. 95% C.I.

Is the preference for sending military aid to governments that align with democratic values more conditional on security threats for some Americans than others? As discussed, internationalists should show a stronger preference than isolationists for aiding democracies with good human rights records, but this preference may also be more sensitive to terrorism concerns, given the tension in their desire for US foreign policy to promote both democratic values and global security. To evaluate this argument, I repeat the conditional analysis of the democratic values treatment effects from the previous section, but this time with the internationalist and isolationist subgroups. Figure 3 displays the results. The top panel reports the democratic values treatment effects for internationalists and isolationists when the terrorist group is active and when it is not. The bottom panel shows the differences between these effects by plotting the interactions between the democratic values and terrorism treatments.

In the combined data and in three of the four surveys, the results show that in the absence of security threats, the democratic values effect is larger among internationalists (21, 35, 30, and 25 percentage points) relative to isolationists (19, 26, 19, and 16 percentage points).^9^ However, the results also show that the magnitude of the democratic values effect declines substantially for internationalists, but not for isolationists, when the terrorism threat is present. In the combined data, the democratic values effect among internationalists declines from 21 percentage points in the absence of the terrorist group to 8 percentage points when the terrorist group is active (*p* < .001). In the first survey, the effect declines from 35 percentage points to 14 percentage points (*p* < .05), in the second survey it falls from 30 to 14 percentage points (*p* < .10), and in the third survey it drops from 25 to 5 percentage points (*p* < .01).

By contrast, the effect of the democratic values treatment among isolationists barely shifts in response to the security threat in the combined data or in the first three surveys. In the combined data, isolationists are 19 percentage points more likely to support aiding the government that reflects democratic values, and this effect barely shifts to 15 percentage points when the terrorist group is present. In the first survey, the democratic values effect for isolationist respondents is 26 percentage points regardless of the threat treatment. In the second survey, the security threat strengthens the democratic values effect by 2 percentage points, and in the third survey it reduces it by only 3 percentage points.

The results are somewhat different in the fourth iteration of the experiment, which was implemented in 2024. Internationalist respondents are 15 percentage points more likely to favor aiding the government that aligns with democratic values in the absence of security threats, but isolationists actually show a slightly stronger reaction, with an 18 percentage point increase for the democratic values treatment without the terrorist threat. The terrorist threat still reduces the democratic values effect more for internationalists, with a decline of 9 percentage points, but this reduction is only slightly larger than that experienced by isolationists, which is 8 percentage points.

Altogether, the threat treatment reduces the magnitude of the democratic values effect by 9 percentage points more for internationalists than isolationists in the combined data (*p* < .05), by 21 percentage points in the first survey (*p* < .10), by 18 percentage points in the second survey (*p* = .18), and by 17 percentage points in the third survey (*p* = .11), whereas the decline is similar among internationalists and isolationists in the fourth iteration of the experiment.

Why did internationalists and isolationists react similarly in the fourth survey? One possibility is that the difference reflects the composition of the sample. The fourth survey included an age quota in which half of the sample was intended to be Americans between the ages of 18 and 30 due to another module on the survey in which it was important to oversample younger adults. However, comparisons of internationalists and isolationists by age group return similar results, suggesting this possibility is unlikely.^10^ Another explanation could be that the types of Americans holding internationalist and isolationist views have changed as American politics shifted under Presidents Trump and Biden. Exploratory analysis suggests this explanation may be part of the story. The partisan and demographic characteristics of the two groups remain relatively constant over the four surveys. However, the interaction between the democratic values and threat treatments are more polarized by educational attainment in the fourth survey relative to the combined data, which could reflect the increasing important of educational polarization in US politics.^11^

However, in general, the results across multiple surveys are consistent with the claim that internationalists are more likely than isolationists to demonstrate a weakened commitment to aiding governments aligned with democratic values when security threats become salient. In Supplemental Information-4.5, I show the average level of support for sending military aid in each of the treatment conditions for both internationalists and isolationists. Regardless of whether the terrorist group was present or not, a sizeable majority of internationalists in all four surveys supported sending military aid to the governments aligned with democratic values. However, the terrorism threat increases internationalist support for aiding the rights-violating autocracy much more than it increases support for the rights-respecting democracy in all four surveys, such that the internationalists’ relative preference for aiding the democracy with the good human rights record shrinks substantially. One concern may be that these results reflect a ceiling effect on internationalists’ support for sending military aid to governments aligned with democratic values. However, there remains room for internationalists to become more supportive of aiding these governments under the terrorist condition in the four surveys.^12^ In Supplemental Information-4, I also show that results are similar when using the ordinal outcome, alternative measures of internationalism on the second and fourth surveys, with the disaggregated democracy and human rights treatments on the fourth survey, and with control variables, including for partisanship, incorporated in the regression models.

### Foreign Policy Orientation and Support for Military Aid

Are internationalists strong but fickle proponents of aligning US foreign policy with democratic values, and does this stance have implications for their attitudes toward military aid? I explore this argument further through descriptive survey data acquired in 2019, 2021, 2023, and 2024, analyzing how foreign policy orientation correlates with support for military aid, democracy promotion, and anti-terrorism partnerships with both authoritarian and democratic governments. I anticipate that internationalists will be simultaneously more likely to support democracy promotion and working with autocracies to fight terrorism, while also being the group most likely to approve of aiding foreign militaries. This constellation of views would help to explain the strength of their preference for sending military aid to governments aligned with democratic values rather than rights-abusing autocracies, but also the sensitivity of this preference to security threats like terrorism.

**Figure 4.**
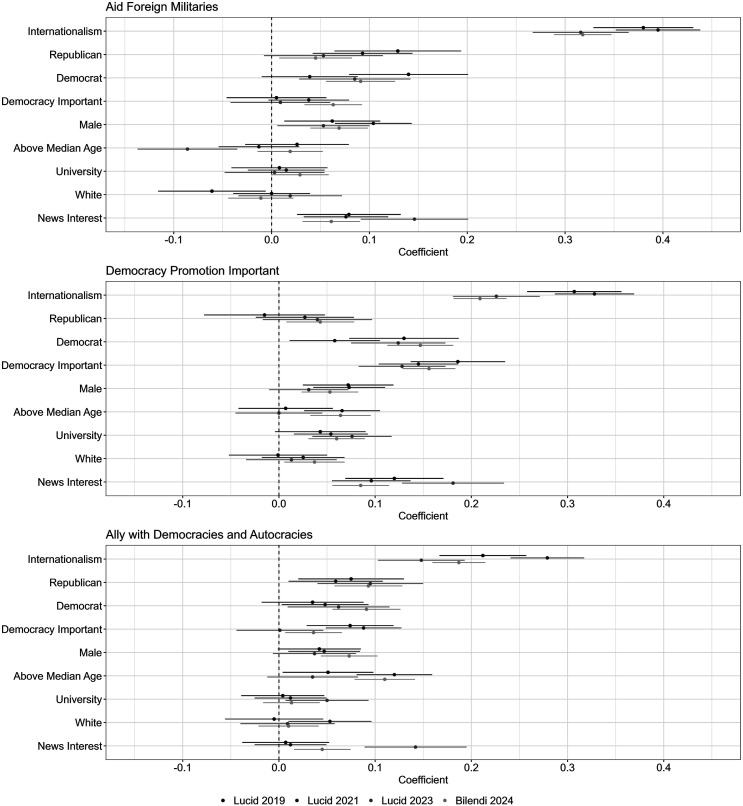
Internationalists simultaneously support military aid, democracy promotion, and anti-terrorism partnerships with autocracies. *Note.* Outcomes are binary indicators for supporting (1) the training and equipping of friendly military forces, (2) democracy promotion as a key focus of US foreign policy, and (3) aligning with any democratic or authoritarian government to fight terrorism abroad. 95% C.I.

For each of the four surveys on which the descriptive questions were asked, I regressed the three outcomes on a dummy for internationalism, along with several other variables that may affect foreign policy attitudes. These include binary variables for alignment with the Republican or Democratic parties, whether they view democracy as important, being male, white, and above median age, having a university degree, and paying close attention to current affairs. The results are displayed in Figure 4. The plot on the top shows the correlates of supporting military aid, the middle plot shows the correlates of wanting democracy promotion to be a key part of US foreign policy, and the plot on the bottom shows the correlates of supporting anti-terrorism partnerships with both autocratic and democratic governments.

For all three outcomes, an internationalist orientation is consistently a strong predictor of support, including relative to partisanship and attitudes toward democracy generally. Across the four surveys, internationalists are 38, 40, 32 and 32 percentage points more likely to favor equipping and training foreign militaries. They are also 31, 33, 23, and 21 percentage points more likely to say that democracy promotion should be an important part of US foreign policy, and they are 21, 28, 15, and 19 percentage points more likely to say the US government should align with both democracies and autocracies to fight terrorism. These substantial relationships contrast with relatively weak partisan effects. Republicans and Democrats are both more likely than independents to support military aid, but they show relatively little difference between them, which appears to set attitudes toward military assistance apart from attitudes toward other types of foreign aid. Democrats are somewhat more likely than both Republicans and independents to favor democracy promotion, and members of both parties are again somewhat more likely than independents to support anti-terrorism partnerships with both democratic and authoritarian governments. The other important correlate is interest in current affairs, especially in the third survey, which may reflect its implementation after the start of the Ukraine war.

What percentage of internationalists hold these views simultaneously? Across the three surveys, 73, 75, 74, and 69 percent of internationalist respondents expressed support for both democracy promotion and working with autocracies to fight terrorism. Of these internationalists, 83, 81, 80, and 85 percent also supported military aid. In other words, large and similar majorities of internationalists in all four surveys held potentially contradictory views in which they favor democracy promotion but also support working with authoritarian governments to contain terrorism threats. The internationalists who held these views simultaneously were also the survey respondents most likely to approve of the United States equipping and training friendly foreign militaries. Furthermore, in Supplemental Information-4.8, I show that these results are consistent for internationalists with more militant or cooperative tendencies, suggesting that this divide is most pronounced between isolationists and internationalists generally. These results align with the argument that internationalist Americans will show a particularly strong preference for sending military aid to democracies over autocracies, but will also be pulled toward conditioning this preference on the salience of security threats like terrorism.

## Discussion

This study explores how alignment with democratic values and the presence of terrorism threats influence Americans’ attitudes toward military aid for foreign governments. The experimental findings reflect previous research demonstrating that Americans’ views of foreign policy are shaped by a preference for partnerships with democratic governments that respect human rights. Across four surveys, the democratic values treatment increased support for military assistance significantly more than the security threat treatment. I also demonstrate that, in the full samples, the strength of the democratic values treatment declines when the terrorist threat is present; however, it remains substantively meaningful, suggesting that terrorism concerns do not overturn the public's preference for aid recipients to uphold democratic values. These results align with previous studies showing that concerns about democracy and human rights can affect the public’s attitudes toward aid more than direct security and economic concerns (Allendoerfer 2017; Doherty et al. 2020). Furthermore, they show that this holds true even for a security-oriented policy like military assistance.

However, I also find evidence that foreign policy orientation moderates how democratic values and terrorist threats interact in shaping attitudes toward military aid. In the combined data and in three of the four experimental studies, internationalist respondents were substantially more sensitive to terrorism threats, which significantly increased their willingness to aid rights-violating autocracies and reduced the relative preference for sending assistance to rights-respecting democracies. Descriptive survey data supports the argument that this reaction can be explained at least in part by internationalist support for a hegemonic United States that seeks to make the world more democratic while also actively managing global security challenges like terrorism. When these two objectives conflict, the results in this study suggest that many internationalists will prioritize security interests like terrorism. They may place relatively more emphasis on alignment with democratic values in aid relationships when security threats are not salient, but their support for proactive US responses to international security reduces their relative preference for aiding governments that uphold democratic values when threats are present.

A possible alternative explanation for these internationalist reactions is that internationalists perceive military aid as a useful tool for *encouraging* democratization and respect for human rights. Though the evidence seems to align more with the argument that US military aid worsens or at least fails to improve either of these outcomes, some scholars have argued that US military aid can facilitate more democratic developments (Atkinson 2014), and research suggests this may be the case for specific training programs like IMET (Omelicheva et al. 2017). Regardless, this explanation for internationalists’ attitudes cannot account for the *conditionality* of their reactions. If they perceive military aid as a useful tool for promoting democratic values, it is less clear why they would demonstrate such a large preference for sending aid to the democracy with the good human rights record when the threatening terrorist group was not present.

As discussed, Americans may want the United States to prioritize partnerships with governments that reflect democratic values not because they view them as morally better, but because they believe these governments are more likely to promote US security interests internationally (Brancati 2014). To some extent, the experimental design accounts for this possibility by stating explicitly in the vignette that the country is a reliable partner of the United States. Democracies may be viewed as more beneficial to US security because they are more likely to align with US foreign policy and because their domestic politics are less likely to cause global disruptions (Wollack 2008). Holding reliability constant in the experiment should reduce the likelihood that respondents focused on these benefits of democratic values when reading the vignette, and it may also have reduced the magnitude of the democratic values effect as a result. Nonetheless, it is entirely plausible that respondents would still believe that governments aligned with democratic values are better for US security interests, and that this belief explains at least part of the democratic values effect. Indeed, those who advocate for prioritizing democratic values in international affairs – including for military aid policies – often supplement moral arguments with claims that this approach would create a friendlier and more stable international system for the United States. To the extent that this view is widespread, it would not undermine the central conclusions that Americans demonstrate a strong preference for military aid recipients to align with democratic values and that this preference is robust to the presence of terrorism threats. People prefer democracy and rights at home and abroad for many reasons, and regardless of their specific motivations, it is useful to understand whether and for whom the importance of these values weakens as threats like terrorism become salient.

Another question about interpretation of the experimental results pertains to the combination of democracy and human rights in the democratic values treatment. As was already discussed, democracies definitionally protect core political freedoms more than autocracies, so mentioning both regime type and rights in the treatment is conceptually useful for assessing the effects of the recipient government’s alignment with democratic values. However, though democracies generally perform better than autocracies at protecting human rights, democratic governments can also engage in severe rights violations. Several top recipients of US military aid are governments that are often classified as (flawed) democracies that frequently violate human rights, including Afghanistan prior to 2021, Iraq, Israel, and Lebanon. Are the violations committed by these governments and their security forces likely to trigger negative reactions to military aid among the American public, even if their governments can arguably be classified as democracies? The disaggregated treatments in the fourth iteration of the experiment may shed some light on this question. The effect of the good human rights treatment is somewhat larger than the effect of the democracy treatment, which could indicate that rights violations will drive negative reactions to military aid even in cases where the recipient government can plausibly be classified as democratic. However, the design does not test this directly, since respondents may infer that countries with good human rights records are democracies and countries with bad human rights records are autocracies (Dafoe et al. 2018). As a result, future work could explore reactions to military aid for these specific cases of rights-violating democracies.

The internationalist reaction to security threats has important implications for understanding the limits of democratic values in shaping US foreign policy. Not only have majorities of Americans expressed an internationalist orientation for decades (Smeltz et al. 2023), but internationalists have dominated the US foreign policy community as well. These internationalists have often been at the forefront of advocating for a US foreign policy that promotes democracy in the international system. Yet, to the extent that elite opinion resembles that of the public (Kertzer 2022), the findings suggest that these key foreign policy elites will often be particularly likely to deemphasize democracy and human rights when confronted with perceived security interests like terrorism, even as they express greater commitment to the idea that US foreign policy should advance democratic values abroad. Explanations for why the US government continues to send military aid to abusive autocracies like Egypt’s have emphasized the bureaucratic interests of the US security apparatus, the commercial interests of the weapons industry, path dependency, or the policy preferences of a few foreign policy elites who prioritize security goals like peace between Israel and Egypt (e.g., Bergmann and Schmitt 2021; Hamed 2021; Miller and Sokolsky 2018). While these explanations are almost certainly relevant, my findings also highlight how these policies will be amenable to many of those Americans who are most likely to care that US foreign policy attempts to promote democracy abroad. As a result, for cases where the US government maintains aid relationships with authoritarian and rights-abusing partners in which key security concerns are present, the results suggest that only a small constituency may exist for whom critiques related to democracy and human rights will be persuasive.

This dynamic could be relevant to the ability of human rights messaging to persuade the American public and American officials to prioritize democratic values when security interests are at stake. Prior studies have explored how governments can limit their vulnerability to naming and shaming by human rights advocates when they can frame their actions as responses to security threats (Jetschke 2010; Williamson and Malik 2021). My results suggest similar reactions are likely to be at play in the United States. Thus, human rights advocates may be more successful at criticizing US military aid for abusive autocrats when they can explain how these policy choices undermine direct US security interests such as combating terrorism as well as US values. Future research could test this idea through additional survey experiments.

One question about generalizability is whether a different security issue would be more likely to swamp the democratic values effect. Terrorism has been a highly salient security issue in US foreign policy debates, including in the years in which the experiment was implemented, and it pertains directly to the justifications given for US military aid in many countries. As a result, the issue is relevant for understanding how Americans weigh democratic values against security interests for military aid relationships. However, the salience of terrorism has faded over time. It is possible that a different type of threat – such as the invasion of a US ally by another country – would be more likely to weaken Americans’ preference for aiding the militaries of governments that align with democratic values. Additional research could consider this potential variation.

Finally, the findings also contribute more broadly to research on attitudes toward foreign aid. They suggest that variation in support for military aid relationships may be explained by different factors than those that predict support for developmental aid or humanitarian assistance. As a result, it would be useful for scholars to explore further the drivers of attitudes toward security assistance, given its importance to US foreign policy and international politics.

## Supplemental Material

Supplemental Material - Who Gets the Guns? How Democratic Values and Security Threats Affect American Attitudes Toward Military Aid Supplemental Material for Who Gets the Guns? How Democratic Values and Security Threats Affect American Attitudes Toward Military Aid by Scott Williamson in Journal of Conflict Resolution

Supplemental Material - Who Gets the Guns? How Democratic Values and Security Threats Affect American Attitudes Toward Military Aid Supplemental Material for Who Gets the Guns? How Democratic Values and Security Threats Affect American Attitudes Toward Military Aid by Scott Williamson in Journal of Conflict Resolution

## Data Availability

Data and replication files are available at https://doi.org/10.7910/DVN/ARAKLD (Williamson 2025).

## Notes

1. At the time of writing, the second Trump administration may have ended – at least temporarily – the dominance of internationalist elites in American foreign policy. However, it remains to be seen whether their elite cues will lead to a genuine shift in the American public’s general orientation toward international affairs.

2. For example Egypt, Saudi Arabia, Yemen.

3. As discussed, Americans may prioritize aid for democracies with good human rights records because of a normative commitment to these values and/or because they believe democracies provide the United States with greater strategic benefits.

4. The individual democracy and human rights treatments, along with the combined democracy and human rights treatment, are both substantively and similarly larger than the disaggregated or combined treatment groups in which the recipient government is an autocracy or a state with a bad human rights record. The treatments are not fully crossed, such that there are no combined treatments where democracies have bad human rights records or autocracies have good human rights records. As a result, respondents may have assumed the country’s regime type from its human rights record, or vice versa (Dafoe et al. 2018), and it is not possible to draw firm conclusions about which produces stronger effects. However, the design fulfills its goal by showing that effects in the combined democratic values treatment are not entirely driven by the explicit mention of democracy or human rights only, and that the effects are robust to mentioning each on their own.

5. In the Bilendi 2024 survey, the human rights treatment was instead labeled as Governance, and respondents were randomized into the following groups: The country’s government is democratic/The country’s government has a reputation for respecting human rights/The country’s government is democratic and has a reputation for respecting human rights/The country’s government is authoritarian/The country’s government has a reputation for violating human rights/The country’s government is authoritarian and has a reputation for violating human rights. Results reported in Supplemental Information-4.7.

6. The region was randomized in the first and second surveys but had no effect on the results. As a result, it was dropped from the third replication. It was added again in the fourth replication but again produced no effects. Results with the region effects are reported in Supplemental Information-4.

7. For the data combined across the four surveys, I also include indicators for each survey wave.

8. In the combined data, 62 percent supported sending aid to the democracy with the good human rights record when no threat was present, and 54 percent supported sending aid to the autocracy with the bad human rights record when the terrorist group was active. In the first survey, 61 percent supported sending aid to the democracy when no threat was present, and 49 percent supported sending aid to the autocracy when the terrorist group was active. In the second survey, the numbers were 52 percent and 46 percent. In the third survey, they were 67 percent and 59 percent. And in the fourth survey, they were 63 and 57 percent.

9. The difference is visible but weak in the combined data because of the large sample size in the fourth survey, where internationalists and isolationists reacted somewhat differently than in the other three surveys.

10. Results are reported in Supplemental Information-4.10.

11. Results are reported in Supplemental Information-4.11.

12. On the first survey, 71 percent of internationalists support military aid for the democracy in the absence of the terrorism threat, and this support increases only modestly to 74 percent with the active terrorist group. In the second survey, the threat condition increases support for aiding the democracy from 59 to 69 percent of internationalists, in the third survey it decreases support from 81 to 73 percent, and in the fourth survey it drops from 81 to 79 percent. By contrast, for the autocracy, the security threat increases internationalist support for military aid from 37 to 61 percent in the first survey, from 29 to 55 percent in the second survey, from 56 to 68 percent in the third survey, and from 65 to 73 percent in the fourth survey.

## Appendix

### Main Effects of Democratic Values and Threat Treatments

Table 2 shows regression results from Figure 1.

**Table 2.** Main effects of democratic values and threat treatments

	Combined data	YouGov 2016	YouGov 2017	Lucid 2019	Bilendi 2024
Democratic values	0.16***	0.25***	0.20***	0.15***	0.13***
	(0.01)	(0.03)	(0.03)	(0.03)	(0.02)
Terrorism threat	0.09***	0.13***	0.15***	0.07**	0.07***
	(0.01)	(0.03)	(0.03)	(0.03)	(0.02)
Constant	0.40***	0.33***	0.30***	0.49***	0.48***
	(0.02)	(0.03)	(0.03)	(0.02)	(0.01)
Observations	7,257	1,000	1,000	1,299	3,958

^†^*p* < .10;^ *^*p* < .05; ^**^*p* < .01; ^***^*p* < .001.

OLS regression models.

### Democratic Values Treatment Conditional on Threat Treatment

Table 3 shows regression results from the bottom plot of Figure 2, in which the democratic values treatment is interacted with the terrorism threat treatment.

**Table 3.** Interaction between democratic values and threat treatments

	Combined data	YouGov 2016	YouGov 2017	Lucid 2019	Bilendi 2024
Democratic values	0.21***	0.29***	0.25***	0.22***	0.18***
	(0.02)	(0.04)	(0.05)	(0.04)	(0.02)
Terrorism threat	0.13***	0.17***	0.19***	0.14***	0.11**
	(0.02)	(0.04)	(0.04)	(0.04)	(0.02)
Values x threat	−0.09***	−0.08	−0.07	−0.13*	−0.09**
	(0.02)	(0.06)	(0.07)	(0.05)	(0.03)
Constant	0.38***	0.31***	0.28***	0.45***	0.45***
	(0.02)	(0.03)	(0.03)	(0.03)	(0.02)
Observations	7,257	1,000	1,000	1,299	3,957

^†^*p* < .10;^ *^*p* < .05; ^**^*p* < .01; ^***^*p* < .001.

OLS regression models.

### Treatment Interactions Conditional on Foreign Policy Orientation

Tables 4 and 5 show the regression models in which the treatments are interacted among internationalists and isolationists, shown in the bottom panel of Figure 3.

**Table 4.** Internationalists: Interaction between democratic values and threat treatments

	Combined	YouGov 2016	YouGov 2017	Lucid 2019	Bilendi 2024
Democratic values	0.21***	0.35***	0.30***	0.25***	0.15***
	(0.02)	(0.07)	(0.08)	(0.05)	(0.03)
Terrorism threat	0.13***	0.24***	0.26***	0.12*	0.08*
	(0.02)	(0.07)	(0.07)	(0.05)	(0.03)
Values x threat	−0.13***	−0.21*	−0.16^†^	−0.20**	−0.09*
	(0.03)	(0.09)	(0.09)	(0.07)	(0.04)
Constant	0.47***	0.37***	0.29***	0.56***	0.65***
	(0.03)	(0.05)	(0.05)	(0.04)	(0.02)
Observations	3,365	393	498	710	1,764

^†^*p* < .10;^ *^*p* < .05; ^**^*p* < .01; ^***^*p* < .001.

OLS regression models.

**Table 5.** Isolationists: Interaction between democratic values and threat treatments

	Combined	YouGov 2016	YouGov 2017	Lucid 2019	Bilendi 2024
Democratic values	0.19***	0.26***	0.19*	0.16**	0.18***
	(0.02)	(0.05)	(0.07)	(0.06)	(0.03)
Terrorism threat	0.13***	0.13*	0.12*	0.14*	0.14***
	(0.02)	(0.05)	(0.06)	(0.06)	(0.03)
Values x threat	−0.04	−0.00	0.02	−0.03	−0.08^†^
	(0.03)	(0.08)	(0.09)	(0.08)	(0.04)
Constant	0.33***	0.28***	0.26***	0.34***	0.30***
	(0.02)	(0.04)	(0.05)	(0.04)	(0.02)
Observations	3,891	607	502	589	2,193

^†^*p* < .10;^ *^*p* < .05; ^**^*p* < .01; ^***^*p* < .001.

OLS regression models.

### Descriptive Results

The following tables show the internationalist coefficients in the regression models from Figure 4: support for military aid; support for democracy promotion; and support for working with autocratic governments against terrorism. Full regression results with all control variables are shown in Supplemental Information-5.

**Table 6.** Internationalism and support for military aid

	Lucid 2019	Lucid 2021	Lucid 2023	Bilendi 2024
Internationalist	0.38***	0.40***	0.32***	0.32***
	(0.03)	(0.02)	(0.03)	(0.02)
Constant	0.23***	0.20***	0.24***	0.26***
	(0.04)	(0.03)	(0.03)	(0.02)
Observations	1,299	1,983	1,532	3,912
Controls	*✓*	*✓*	*✓*	*✓*

^†^*p* < .10;^ *^*p* < .05; ^**^*p* < .01; ^***^*p* < .001.

OLS regression models.

**Table 7.** Internationalism and support for democracy promotion

	Lucid 2019	Lucid 2021	Lucid 2023	Bilendi 2024
Internationalist	0.31***	0.33***	0.23***	0.21***
	(0.03)	(0.02)	(0.02)	(0.01)
Constant	0.22***	0.22***	0.31***	0.28***
	(0.03)	(0.03)	(0.03)	(0.02)
Observations	1,299	1,984	1,532	3,912
Controls	*✓*	*✓*	*✓*	*✓*

^†^*p* < .10;^ *^*p* < .05; ^**^*p* < .01; ^***^*p* < .001.

OLS regression models.

**Table 8.** Internationalism and support for working with autocrats

	Lucid 2019	Lucid 2021	Lucid 2023	Bilendi 2024
Internationalist	0.21***	0.28***	0.15***	0.19***
	(0.02)	(0.02)	(0.02)	(0.01)
Constant	0.56***	0.37***	0.47***	0.41***
	(0.03)	(0.03)	(0.03)	(0.02)
Observations	1,299	1,981	1,532	3,912
Controls	*✓*	*✓*	*✓*	*✓*

^†^*p* < .10;^ *^*p* < .05; ^**^*p* < .01; ^***^*p* < .001.

OLS regression models.

## References

[bibr1-00220027251388634] Ackerman Spencer . 2015. “Obama Restores US Military Aid to Egypt over Islamic State Concerns.” The Guardian; March 31.

[bibr2-00220027251388634] Albertus Michael Menaldo Victor . 2012. “Coercive Capacity and the Prospects for Democratization.” Comparative Politics 44 (2): 151–169.

[bibr3-00220027251388634] Allendoerfer Michelle Giacobbe . 2017. “Who Cares About Human Rights? Public Opinion About Human Rights Foreign Policy.” Journal of Human Rights 16 (4): 428–451.

[bibr4-00220027251388634] Anderson Stephanie B. Garrison Jean A . 2024. “The Public’s Commitment Toward US Leadership of the Liberal International Order.” In Polarization and Deep Contestations: The Liberal Script in the United States, edited by Börzel Tanja A. Risse Thomas Anderson Stephanie B. Garrison Jean A. . Oxford University Press.

[bibr5-00220027251388634] Atkinson Carol . 2014. Military Soft Power: Public Diplomacy Through Military Educational Exchanges. Bloomsbury Publishing PLC.

[bibr6-00220027251388634] Bellin Eva . 2004. “The Robustness of Authoritarianism in the Middle East: Exceptionalism in Comparative Perspective.” Comparative Politics 36 (2): 139–157.

[bibr7-00220027251388634] Bergmann Max Schmitt Alexandra . 2021. “A Plan to Reform U.S. Security Assistance.” Center for American Progress; March 9.

[bibr8-00220027251388634] Blackman Alexandra Domike . 2018. “Religion and Foreign Aid.” Politics and Religion 11 (3): 522–552.

[bibr9-00220027251388634] Boutton Andrew Carter David B . 2014. “Fair-Weather Allies? Terrorism and the Allocation of US Foreign Aid.” Journal of Conflict Resolution 58 (7): 1144–1173.

[bibr10-00220027251388634] Brancati Dawn . 2014. “The Determinants of US Public Opinion Towards Democracy Promotion.” Political Behavior 36 (4): 705–730.

[bibr83-00220027251388634] Busby Joshua W. Monten Jonathan . 2008. “Without Heirs? Assessing the Decline of Establishment Internationalism in U.S. Foreign Policy.” Perspectives on Politics 6 (3): 451–472.

[bibr11-00220027251388634] Bush Sarah Sunn Zetterberg Pär . 2021. “Gender Quotas and International Reputation.” American Journal of Political Science 65 (2): 326–341.

[bibr12-00220027251388634] Carriere Kevin R. Hallahan Anna Moghaddam Fathali M . 2022. “The Effect of Perceived Threat on Human Rights: A Meta-Analysis.” Group Processes & Intergroup Relations 25 (1): 247–279.

[bibr13-00220027251388634] Chanley Virginia A. 1999. “U.S. Public Views of International Involvement from 1964 to 1993: Time-Series Analyses of General and Militant Internationalism.” Journal of Conflict Resolution 43 (1): 23–44.

[bibr14-00220027251388634] Chaudoin Stephen Milner Helen V. Tingley Dustin H . 2010. “The Center Still Holds: Liberal Internationalism Survives.” International Security 35 (1): 75–94.

[bibr15-00220027251388634] Chu Jonathan A. Ko Jiyoung Liu Adam . 2021. “Commanding Support: Values and Interests in Rhetoric of Alliance Politics.” International Interactions 47 (3): 477–503.

[bibr16-00220027251388634] Chu Jonathan A. Williamson Scott Yeung Eddy S.F . 2024. “People Consistently View Elections and Civil Liberties as Key Components of Democracy.” Science 387 (6719): 291–296. 10.1126/science.adp1274 39418359

[bibr17-00220027251388634] Dafoe Allan Zhang Baobao Caughey Devin . 2018. “Information Equivalence in Survey Experiments.” Political Analysis 26 (4): 399–416.

[bibr18-00220027251388634] Dasandi Niheer Fisher Jonathan Hudson David vanHeerde-Hudson Jennifer . 2022. “Human Rights Violations, Political Conditionality, and Public Attitudes to Foreign Aid: Evidence from Survey Experiments.” Political Studies 70 (3): 603–623.

[bibr19-00220027251388634] Davis Darren W. Silver Brian D . 2003. “Civil Liberties vs. Security: Public Opinion in the Context of the Terrorist Attacks on America.” American Journal of Political Science 48 (1): 28–46.

[bibr87-00220027251388634] Dodson Kyle Brooks Clem . 2022. “All by Himself? Trump, Isolationism, and the American Electorate.” The Sociological Quarterly 63 (4): 780–803.

[bibr20-00220027251388634] Doherty David Bryan Amanda Clare Hanania Dina Pajor Matthew . 2020. “The Public’s Foreign Aid Priorities: Evidence from a Conjoint Experiment.” American Politics Research 48 (5): 635–648.

[bibr21-00220027251388634] Dube Oeindrila Naidu Suresh . 2015. “Bases, Bullets, and Ballots: The Effect of US Military Aid on Political Conflict in Columbia.” The Journal of Politics 77 (1): 249–267.

[bibr22-00220027251388634] Gambino Lauren Borger Julian 2019. “Yemen War: Congress Votes to End US Military Assistance to Saudi Arabia.” *The Guardian*; April 4.

[bibr23-00220027251388634] Godefroidt Amélie . 2023. “How Terrorism Does (and Does Not) Affect Citizens’ Political Attitudes: A Meta-Analysis.” American Journal of Political Science 67 (1): 22–38.

[bibr24-00220027251388634] Grewal Sharan . 2022. “Norm Diffusion Through US Military Training in Tunisia.” Security Studies 31 (2): 291–317.

[bibr25-00220027251388634] Hamed Yehia. 2021. “U.S. Military Aid Has Helped Crush Egypt’s Civil Society. End It Now.” *DAWN*; August 23.

[bibr26-00220027251388634] Heinrich Tobias Kobayashi Yoshiharu . 2020. “How Do People Evaluate Foreign Aid to ‘Nasty’ Regimes?” British Journal of Political Science 50 (1): 103–127.

[bibr27-00220027251388634] Heinrich Tobias Kobayashi Yoshiharu Long Leah . 2018. “Voters Get What They Want (When They Pay Attention): Human Rights, Policy Benefits, and Foreign Aid.” International Studies Quarterly 62 (1): 195–207.

[bibr28-00220027251388634] Hetherington Marc Suhay Elizabeth . 2011. “Authoritarianism, Threat, and Americans’ Support for the War on Terror.” American Journal of Political Science 55 (3): 546–560.

[bibr29-00220027251388634] Higgins Martha K. 1998. Political Policing: The United States and Latin America. Duke University Press.

[bibr30-00220027251388634] Holsti Ole R. Rosenau James N . 1990. “The Structure of Foreign Policy Attitudes Among American Leaders.” The Journal of Politics 52 (1): 94–125.

[bibr31-00220027251388634] Hook Steven W. 1998. “Building Democracy Through Foreign Aid: The Limitations of United States Political Conditionalities, 1992-96.” Democratization 5 (3): 156–180.

[bibr32-00220027251388634] Huddy Leonie Feldman Stanley Taber Charles Lahav Gallya . 2005. “Threat, Anxiety, and Support of Antiterrorism Policies.” American Journal of Political Science 49 (3): 593–608.

[bibr33-00220027251388634] Human Rights Watch . 2011. “US: Suspend Military Aid to Yemen.”.

[bibr34-00220027251388634] Human Rights Watch . 2023. “Joint Letter Urging Biden Administration Not to Provide Military Aid to Egypt in Light of Ongoing Human Rights Violations.” July 28.

[bibr35-00220027251388634] Ikenberry G. John . 2009. “Liberal Internationalism 3.0: America and the Dilemmas of Liberal World Order.” Perspectives on Politics 7 (1): 71–87.

[bibr36-00220027251388634] Jetschke Anja . 2010. Human Rights and State Security: Indonesia and the Philippines. University of Pennsylvania Press.

[bibr37-00220027251388634] Johns Robert Davies Graeme A. M . 2012. “Democratic Peace or Clash of Civilizations? Target States and Support for War in Britain and the United States.” The Journal of Politics 74 (4): 1038–1052.

[bibr38-00220027251388634] Joyce Renanah Miles . 2022. “Soldiers’ Dilemma: Foreign Military Training and Liberal Norm Conflict.” International Security 46 (4): 48–90.

[bibr39-00220027251388634] Kertzer Joshua D. 2013. “Making Sense of Isolationism: Foreign Policy Mood as a Multilevel Phenomenon.” The Journal of Politics 75 (1): 225–240.

[bibr40-00220027251388634] Kertzer Joshua . 2022. “Re-Assessing Elite-Public Gaps in Political Behavior.” American Journal of Political Science 66 (3): 539–553.

[bibr94-00220027251388634] Kertzer Joshua D. Powers Kathleen E. Rathbun Brian C. Iyer Ravi . 2014. “Moral Support: How Moral Values Shape Foreign Policy Attitudes.” The Journal of Politics 76 (3): 825–840.

[bibr41-00220027251388634] Kertzer Joshua D. Zeitzoff Thomas . 2017. “A Bottom-Up Theory of Public Opinion About Foreign Policy.” American Journal of Political Science 61 (3): 543–558.

[bibr42-00220027251388634] Lührmann Anna Tannenberg Marcus Lindberg Staffan I. . 2018. “Regimes of the World (RoW): Opening New Avenues for the Comparative Study of Political Regimes.” Politics and Governance 6 (1): 60–77.

[bibr43-00220027251388634] Maggiotto Michael A. Wittkopf Eugene R . 1981. “American Public Attitudes Toward Foreign Policy.” International Studies Quarterly 25 (4): 601–631.

[bibr44-00220027251388634] Martinez Machain Carla . 2021. “Exporting Influence: U.S. Military Training as Soft Power.” Journal of Conflict Resolution 65 (2-3): 313–341.

[bibr45-00220027251388634] McCoy Katherine E . 2005. “Trained to Torture? The Human Rights Effects of Military Training at the School of the Americas.” Latin American Perspectives 32 (6): 47–64.

[bibr46-00220027251388634] Miller Andrew Sokolsky Richard . 2018. “What Has $49 Billion in Foreign Military Aid Bought Us? Not Much.” *American Conservative*; February 27.

[bibr48-00220027251388634] Milner Helen V. Tingley Dustin . 2013. “Public Opinion and Foreign Aid: A Review Essay.” International Interactions 39 (3): 389–401.

[bibr47-00220027251388634] Milner Helen V. Tingley Dustin . 2015. Sailing the Water’s Edge: The Domestic Politics of American Foreign Policy. Princeton University Press.

[bibr49-00220027251388634] Møller Jørgen Skaaning Svend-Erik . 2013. “Autocracies, Democracies, and the Violation of Civil Liberties.” Democratization 20 (1): 82–106.

[bibr50-00220027251388634] Nielsen Richard A. 2013. “Rewarding Human Rights? Selective Aid Sanctions Against Repressive States.” International Studies Quarterly 57 (4): 791–803.

[bibr82-00220027251388634] Office of Security Assistance . 2023. “What We Do.” US Department of State, Bureau of Political-Military Affairs.

[bibr51-00220027251388634] Omelicheva Mariya Carter Brittnee Campbell Luke B . 2017. “Military Aid and Human Rights: Assessing the Impact of U.S. Security Assistance Programs.” Political Science Quarterly 132 (1): 119–144.

[bibr52-00220027251388634] Page Benjamin I. Barabas Jason . 2000. “Foreign Policy Gaps Between Citizens and Leaders.” International Studies Quarterly 44 (3): 339–364.

[bibr53-00220027251388634] Powers Ryan Renshon Jonathan . 2021. “International Status Concerns and Domestic Support for Political Leaders.” American Journal of Political Science 67 (3): 732–747.

[bibr54-00220027251388634] Powers Kathleen E. Kertzer Joshua D. Brooks Deborah J. Brooks Stephen G . 2022. “What’s Fair in International Politics? Equity, Equality, and Foreign Policy Attitudes.” Journal of Conflict Resolution 66 (2): 217–245.

[bibr55-00220027251388634] Prather Lauren . 2024. “Ideology at the Water’s Edge: Explaining Variation in Public Support for Foreign Aid.” World Development 176: 106472.

[bibr56-00220027251388634] Rabe Stephen G. 2012. The Killing Zone: The United States Wages Cold War in Latin America. Oxford University Press.

[bibr58-00220027251388634] Rousseau David L. 2005. Democracy and War: Institutions, Norms, and the Evolution of International Conflict. Stanford University Press.

[bibr59-00220027251388634] Ruby Tomislav Z. Gibler Douglas . 2010. “US Professional Military Education and Democratization Abroad.” European Journal of International Relations 16 (3): 339–364.

[bibr60-00220027251388634] Sandholtz Wayne . 2016. “United States Military Assistance and Human Rights.” Human Rights Quarterly 38 (4): 1070–1101.

[bibr61-00220027251388634] Savage Jesse Dillon Caverley Jonathan D . 2017. “When Human Capital Threatens the Capitol: Foreign Aid in the Form of Military Training and Coups.” Journal of Peace Research 54 (4): 542–557.

[bibr62-00220027251388634] Scahill Jeremy . 2012. “Washington’s War in Yemen Backfires.” The Nation; February 15.

[bibr63-00220027251388634] Shapiro Robert Y. Page Benjamin I . 1988. “Foreign Policy and the Rational Public.” Journal of Conflict Resolution 32 (2): 211–247.

[bibr88-00220027251388634] Smeltz Dina Friedhoff Karl Kafura Craig El Baz Lama Berry Libby . 2023. “A Cost of Conflict: Americans Turn Inward.” The Chicago Council on Global Affairs.

[bibr64-00220027251388634] Stoll Richard J. Eichenberg Richard C. Lizotte Mary-Kate . 2023. “The Impact of Personal Security Dispositions on Citizen Support for the Pursuit of Gender Equality in US Foreign Policy.” Journal of Conflict Resolution 67 (5): 923–950.

[bibr65-00220027251388634] Sullivan Patricia L. 2023. “Lethal Aid and Human Security: The Effects of US Security Assistance on Civilian Harm in Low- and Middle-Income Countries.” Conflict Management and Peace Science 40 (5): 467–488.

[bibr66-00220027251388634] Sullivan Patricia L. Tessman Brock F. Li Xiaojun . 2011. “US Military Aid and Recipient State Cooperation.” Foreign Policy Analysis 7 (3): 275–294.

[bibr67-00220027251388634] Tomz Michael R. Weeks Jessica L. P . 2013. “Public Opinion and the Democratic Peace.” American Political Science Review 107 (4): 849–865.

[bibr68-00220027251388634] Tomz Michael Weeks Jessica L. P . 2020. “Human Rights and Public Support for War.” The Journal of Politics 82 (1): 182–194.

[bibr69-00220027251388634] Williamson Scott , 2025. “Replication Data for: Who Gets the Guns? How Democratic Values and Security Threats Affect American Attitudes toward Military Aid.” doi:10.7910/DVN/ARAKLD, Harvard Dataverse, V1. PMC13211145 42206187

[bibr70-00220027251388634] Williamson Scott Malik Mashail . 2021. “Contesting Narratives of Repression: Experimental Evidence from Sisi’s Egypt.” Journal of Peace Research 58 (5): 1018–1033.

[bibr71-00220027251388634] Wittkopf Eugene R. 1986. “On the Foreign Policy Beliefs of the American People: A Critique and Some Evidence.” International Studies Quarterly 30 (4): 425–445.

[bibr72-00220027251388634] Wollack Kenneth . 2008. “Democracy Promotion: Serving U.S. Values and Interests.” Northwestern University Law Review 102 (1): 433–436.

[bibr73-00220027251388634] Zvobgo Kelebogile . 2019. “Human Rights Versus National Interests: Shifting US Public Attitudes on the International Criminal Court.” International Studies Quarterly 63 (4): 1065–1078.

