# Supplementary material for: Who Gets the Guns? How Democratic Values and Security Threats Affect American Attitudes Toward Military Aid
Source: J Conflict Resolut. 2025 Oct 28;70(6):986–1017. doi: 10.1177/00220027251388634 (PMC13211145; doi:10.1177/00220027251388634)
Supplement: Supplemental Material - Who Gets the Guns? How Democratic Values and Security Threats Affect American Attitudes Toward Military Aid [file sj-pdf-1-jcr-10.1177_00220027251388634.pdf]

Supplementary Information

Who Gets the Guns? How Democratic Values and Security Threats Affect American Attitudes toward Military Aid

October 2, 2025

Abstract

The United States gives substantial aid to the militaries of autocratic governments that abuse human rights. US officials claim this aid is necessary to manage security threats, but others argue the United States should prioritize aid for governments that reflect democratic values. How do these competing concerns shape Americans' attitudes toward military aid? Through an experiment implemented on four surveys, I document a strong preference for aiding democracies that respect human rights, and this preference is robust to the presence of terrorism threats. However, internationalist Americans become especially less likely to prioritize democratic values when terrorist threats exist. Descriptive survey questions reinforce this pattern by showing how internationalists who support military aid the most are conflicted proponents of prioritizing democratic values in US foreign policy. The article extends research on attitudes toward foreign aid and illustrates an important limitation to the influence of democratic values on Americans' foreign policy preferences.

Contents

1 Surveys **2**

2 Balance Tests **3**

3 Question Wording **4**

3.1 Prompt 4
3.2 Outcome 4
3.3 Other Variables 4

4 Experimental Results **6**

4.1 Results from Combined Survey Data 6
4.2 Main Effects 7
4.3 Main Effects with Controls 8
4.4 Democratic Values Effects Conditional on Threat 9
4.5 Support by Treatment Group and Foreign Policy Orientation 11
4.6 Conditional Democracy Effects by Foreign Policy Orientation 11
4.7 Disaggregated Regime Type and Human Rights Effects 16
4.8 Effects with Militant and Cooperative Internationalists 18
4.9 Descriptive Characteristics of Internationalists and Isolationists by Survey . 19
4.10 Effects by Foreign Policy Orientation and Age 20
4.11 Democratic Values, Threat, and Education 21

5 Descriptive Results **22**

5.1 Support for Military Aid 22
5.2 Support for Democracy Promotion 23
5.3 Support for Working with Autocrats against Terrorism 24
5.4 Descriptive Analysis of Militant and Cooperative Internationalists 25

1 Surveys

- YouGov 2016 (Survey 1): The sample was collected by YouGov, which also programmed and implemented the survey on its platform. The sample included quotas matched to parameters of the American adult population on gender, age, race, education, party identification, ideology, and political interest. The sample size was 1,000 respondents, and the survey ran from December 27, 2016 to January 3, 2017.

- YouGov 2017 (Survey 2): The sample was collected by YouGov, which also programmed and implemented the survey on its platform. The sample included quotas matched to parameters of the American adult population on gender, age, race, education, party identification, ideology, and political interest. The sample size was 1,000 respondents, and the survey ran from April 6, 2017 to April 17, 2017.

- Lucid 2019 (Survey 3): The sample was collected by Lucid Theorem, and the survey was implemented on Qualtrics. The sample included quotas matched to parameters of the American adult population on gender, age, race, education, party identification, income, and region. The sample size was 1,299, and the survey ran from April 14, 2019 to April 15, 2019.

- Lucid 2021 (Survey 4): The sample was collected by Lucid Theorem, and the survey was implemented on Qualtrics. The sample included quotas matched to parameters of the American adult population on gender, age, race, education, party identification, income, and region. The sample size was 1,983, and the survey ran from May 30, 2021 to June 2, 2021.

- Lucid 2023 (Survey 5): The sample was collected by Lucid Theorem, and the survey was implemented on Qualtrics. The sample included quotas matched to parameters of the American adult population on gender, age, race, education, party identification, income, and region. The sample size was 1,532, and the survey ran from July 2, 2023 to July 19, 2023.

- Bilendi 2024 (Survey 6): The sample was collected by Bilendi, and the survey was implemented on Qualtrics. The survey oversamples young adults between the ages of 18 and 30, with approximately half of respondents in this age range. It included quotas matched to parameters of the US adult population on gender and region. The sample size was 3,957, and the survey ran from December, 12, 2024 to December 28, 2024.

2 Balance Tests

Table 1: *Balance Tests for Democracy and Threat Randomizations*

	YouGov 2016 Dem	YouGov 2016 Threat	YouGov 2017 Dem	YouGov 2017 Threat	Lucid 2019 Dem	Lucid 2019 Threat	Bilendi 2024 Dem	Bilendi 2024 Threat
Male	-	-	†	†	-	-	-	†
Education	†	-	-	-	-	-	-	-
Party ID	-	-	-	-	-	-	-	-
Ideology	-	-	-	-				
Religion	-	-	-	-			*	-
Income	-	-	-	†	-	-	-	-
Race	-	-	-	-	†	-	-	-
News Interest	-	-	†	-	-	-	-	-
Internationalist	-	-	-	-	-	†	-	-

- $p>0.10$; $^{\dagger}p<0.10$; $^{*}p<0.05$; $^{**}p<0.01$; $^{***}p<0.001$
T-tests by treatment status for each variable.

3 Question Wording

3.1 Prompt

As part of its foreign policy, the United States often provides financial and technical support to the militaries of friendly foreign governments. This support can contribute to US foreign policy objectives by helping to maintain close relationships with these governments. However, the US government has limited resources and must choose carefully whether a country should receive this support. Consider a common situation in which US officials are debating whether to fund and train the military of a country in [Africa/the Middle East/Asia]. Some details about the country and its government are provided below. Please read these details carefully and then answer the questions.

- **Reliability:** The country's government has been a reliable partner of the United States.
- **Military:** The country has a large but poorly trained military that would benefit from US assistance.
- **Human Rights:** The country's government is democratic and has a reputation for respecting human rights / The country's government is authoritarian and has a reputation for violating human rights.
- **US Security:** There are no direct threats to US security in this country / The country's military is currently fighting a terrorist group that has attacked the United States and killed dozens of American citizens / (Second survey only) The country's military is currently fighting a terrorist group that has attacked the United States and killed hundreds of American citizens.

3.2 Outcome

Support Aid: Based on the information you just read, do you agree or disagree that the US government should fund and train this country's military?" (strongly agree, agree, somewhat agree, neither agree nor disagree, somewhat disagree, disagree, strongly disagree)

3.3 Other Variables

Internationalist: Do you agree or disagree that the United States should take a more active role in world affairs? (agree strongly, agree somewhat, agree slightly, disagree slightly, disagree somewhat, disagree strongly)[1]

Alternative Internationalist (Third Survey Only): Do you think it will be best for the future of the United States if we take an active part in world affairs or if we stay out of

[1] These answer choices were used for the first two surveys. In the third survey, answer choices ranged from strongly agree to strongly disagree on a seven point scale that included an option for 'neither agree nor disagree.' Ambivalent respondents were included with the isolationists.

world affairs? (Active part in world affairs, Stay out of world affairs)

Partisanship: Do you consider yourself to be a Democrat, a Republican, an independent, or none of these? (Democrat, Republican, Independent, None of the above, Don't know)

Military Aid (Descriptive Analysis): The United States should train and equip the militaries of friendly foreign governments as part of its national security policy. (strongly agree, agree, somewhat agree, neither agree nor disagree, somewhat disagree, disagree, strongly disagree)

Democracy Promotion in US Foreign Policy (Descriptive Analysis): The United States should promote democracy and democratic values abroad as part of its foreign policy. (strongly agree, agree, somewhat agree, neither agree nor disagree, somewhat disagree, disagree, strongly disagree)

Work with Autocrats (Descriptive Analysis): The United States should work with all foreign governments that are willing to help us fight terrorism, including both democratic and authoritarian governments. (strongly agree, agree, somewhat agree, neither agree nor disagree, somewhat disagree, disagree, strongly disagree)

4 Experimental Results

4.1 Results from Combined Survey Data

Table 2: *Main Effects and Conditional Democratic Values Effects with Combined Data*

	Binary Full	Binary No Threat	Binary Threat	Binary Full	Ordinal Full	Ordinal No Threat	Ordinal Threat	Ordinal Full
Democratic Values	0.16***	0.21***	0.12***	0.21***	0.62***	0.73***	0.52***	0.74***
	(0.01)	(0.02)	(0.02)	(0.02)	(0.04)	(0.06)	(0.05)	(0.06)
Terrorism Threat	0.09***			0.13***	0.33***			0.44***
	(0.01)			(0.02)	(0.04)			(0.06)
Values x Threat				-0.09***				-0.22**
				(0.02)				(0.08)
Constant	0.40***	0.36***	0.53***	0.38***	4.04***	3.94***	4.47***	3.99***
	(0.02)	(0.02)	(0.02)	(0.02)	(0.06)	(0.07)	(0.08)	(0.06)
Observations	7,257	3,447	3,810	7,257	7,256	3,447	3,809	7,256
Survey Dummies	✓	✓	✓	✓	✓	✓	✓	✓

$^{\dagger}p<0.10; ^{*}p<0.05; ^{**}p<0.01; ^{***}p<0.001$
OLS regression models.

Table 3: *Internationalist and Isolationist Effects with Combined Data*

	Binary Internationalist	Binary Isolationist	Ordinal Internationalist	Ordinal Isolationist
Democratic Values	0.21***	0.19***	0.73***	0.71***
	(0.02)	(0.02)	(0.08)	(0.07)
Terrorism Threat	0.13***	0.13***	0.40***	0.45***
	(0.02)	(0.02)	(0.08)	(0.07)
Values x Threat	-0.13***	-0.04	-0.32**	-0.12
	(0.03)	(0.03)	(0.10)	(0.10)
Constant	0.47***	0.33***	4.37***	3.75***
	(0.03)	(0.02)	(0.09)	(0.08)
Observations	3,365	3,891	3,364	3,891
Survey Dummies	✓	✓	✓	✓

$^{\dagger}p<0.10; ^{*}p<0.05; ^{**}p<0.01; ^{***}p<0.001$
OLS regression models.

4.2 Main Effects

Table 4: *Main Effects of Democratic Values and Threat Treatments*

	Combined Data	YouGov 2016	YouGov 2017	Lucid 2019	Bilendi 2024
Democratic Values	0.16***	0.25***	0.20***	0.15***	0.13***
	(0.01)	(0.03)	(0.03)	(0.03)	(0.02)
Terrorism Threat	0.09***	0.13***	0.15***	0.07**	0.07***
	(0.01)	(0.03)	(0.03)	(0.03)	(0.02)
Constant	0.40***	0.33***	0.30***	0.49***	0.48***
	(0.02)	(0.03)	(0.03)	(0.02)	(0.01)
Observations	7,257	1,000	1,000	1,299	3,958

†p<0.10;*p<0.05; **p<0.01; ***p<0.001
OLS regression models.

Table 5: *Main Effects with Ordinal Outcome*

	Combined Data	YouGov 2016	YouGov 2017	Lucid 2019	Bilendi 2024
Democratic Values	0.62***	0.96***	0.74***	0.69***	0.48***
	(0.04)	(0.10)	(0.10)	(0.09)	(0.05)
Terrorism Threat	0.33***	0.42***	0.61***	0.30***	0.25***
	(0.04)	(0.10)	(0.11)	(0.09)	(0.05)
Constant	4.04***	3.83***	3.66***	4.35***	4.31***
	(0.06)	(0.09)	(0.11)	(0.08)	(0.05)
Observations	7,256	1,000	999	1,299	3,958

†p<0.10;*p<0.05; **p<0.01; ***p<0.001
OLS regression models.

4.3 Main Effects with Controls

Table 6: *Main Effects with Controls*

	YouGov 2016	YouGov 2017	Lucid 2019	Bilendi 2024
Democratic Values	0.25***	0.20***	0.15***	0.13***
	(0.03)	(0.03)	(0.03)	(0.02)
Terrorism Threat	0.13***	0.14***	0.07**	0.07**
	(0.03)	(0.03)	(0.03)	(0.02)
Middle East Effect	0.02	-0.01		0.01
	(0.04)	(0.04)		(0.02)
Africa Effect	-0.00	-0.02		-0.00
	(0.04)	(0.04)		(0.02)
Constant	0.09†	0.14**	0.29***	0.34***
	(0.05)	(0.05)	(0.04)	(0.03)
Observations	1,000	1,000	1,299	3,930
Controls	✓	✓	✓	✓

†p<0.10;*p<0.05; **p<0.01; ***p<0.001
OLS regression models. Controls for gender, college, partisanship, age, race, news interest.

Table 7: *Main Effects with Ordinal Outcome and Controls*

	YouGov 2016	YouGov 2017	Lucid 2019	Bilendi 2024
Democratic Values	0.96***	0.74***	0.68***	0.47***
	(0.10)	(0.10)	(0.09)	(0.05)
Terrorism Threat	0.39***	0.57***	0.30***	0.26***
	(0.10)	(0.11)	(0.09)	(0.05)
Middle East Effect	0.05	-0.01		0.05
	(0.12)	(0.13)		(0.06)
Africa Effect	0.06	-0.04		-0.02
	(0.12)	(0.13)		(0.06)
Constant	3.30***	3.39***	4.00***	4.04***
	(0.17)	(0.16)	(0.13)	(0.08)
Observations	1,000	1,000	1,299	3,930
Controls	✓	✓	✓	✓

†p<0.10;*p<0.05; **p<0.01; ***p<0.001
OLS regression models. Controls for gender, college, partisanship, age, race, news interest.

4.4 Democratic Values Effects Conditional on Threat

Table 8: *Interaction between Democratic Values and Threat Treatments with Controls*

	YouGov 2016	YouGov 2017	Lucid 2019	Bilendi 2024
Democratic Values	0.29***	0.24***	0.21***	0.18***
	(0.04)	(0.05)	(0.04)	(0.02)
Terrorism Threat	0.16***	0.17***	0.13**	0.12***
	(0.04)	(0.04)	(0.04)	(0.02)
Values x Threat	-0.08	-0.06	-0.12*	-0.09**
	(0.06)	(0.07)	(0.05)	(0.03)
Middle East Effect	0.02	-0.01		0.01
	(0.04)	(0.04)		(0.02)
Africa Effect	-0.00	-0.02		0.00
	(0.04)	(0.04)		(0.02)
Constant	0.08	0.12*	0.26***	0.31***
	(0.05)	(0.05)	(0.04)	(0.03)
Observations	1,000	1,000	1,299	3,930

†p<0.10;*p<0.05; **p<0.01; ***p<0.001
OLS regression models. Controls for gender, college, partisanship, age, race, news interest.

Table 9: *Interaction with Ordinal Outcome*

	YouGov 2016	YouGov 2017	Lucid 2019	Bilendi 2024
Democratic Values	1.06***	0.87***	0.88***	0.58***
	(0.14)	(0.19)	(0.13)	(0.07)
Terrorism Threat	0.52***	0.70***	0.49***	0.35***
	(0.14)	(0.16)	(0.13)	(0.07)
Values x Threat	-0.20	-0.18	-0.38*	-0.20*
	(0.20)	(0.23)	(0.17)	(0.10)
Constant	3.78***	3.60***	4.25***	4.26***
	(0.10)	(0.13)	(0.09)	(0.05)
Observations	1,000	999	1,299	3,958

†p<0.10;*p<0.05; **p<0.01; ***p<0.001
OLS regression models.

Table 10: *Interaction with Ordinal Outcome and Controls*

	YouGov 2016	YouGov 2017	Lucid 2019	Bilendi 2024
Democratic Values	1.06***	0.85***	0.85***	0.59***
	(0.13)	(0.19)	(0.13)	(0.07)
Terrorism Threat	0.50**	0.65***	0.47***	0.38***
	(0.14)	(0.16)	(0.13)	(0.07)
Values x Threat	-0.21	-0.16	-0.34*	-0.23*
	(0.20)	(0.23)	(0.17)	(0.10)
Middle East Effect	0.04	-0.01		0.06
	(0.12)	(0.13)		(0.06)
Africa Effect	0.06	-0.04		-0.02
	(0.12)	(0.13)		(0.06)
Constant	3.25***	3.34***	3.91***	3.99***
	(0.17)	(0.19)	(0.14)	(0.09)
Observations	1,000	999	1,299	3,930

$^{\dagger}p<0.10$; $^{*}p<0.05$; $^{**}p<0.01$; $^{***}p<0.001$
OLS regression models. Controls for gender, college, partisanship, age, race, news interest.

4.5 Support by Treatment Group and Foreign Policy Orientation

Table 11: *Respondents Supporting Military Aid by Treatment and Foreign Policy Orientation*

	Internationalists		Isolationists	
	Dem and HR	Autoc and No HR	Dem and HR	Autoc and No HR
Terrorism Threat				
Combined	75%	67%	58%	43%
YouGov 2016	74%	60%	67%	41%
YouGov 2017	69%	55%	59%	38%
Lucid 2019	73%	68%	61%	48%
Bilendi 2024	79%	73%	54%	44%
No Direct Threat				
Combined	76%	56%	49%	30%
YouGov 2016	71%	37%	54%	28%
YouGov 2017	59%	29%	45%	26%
Lucid 2019	81%	56%	50%	34%
Bilendi 2024	81%	65%	48%	30%

4.6 Conditional Democracy Effects by Foreign Policy Orientation

Table 12: *YouGov 2016: Internationalists and Isolationists*

	Inter No Threat	Inter Threat	Iso No Threat	Iso Threat
Democratic Values	0.35***	0.14*	0.26***	0.26***
	(0.07)	(0.07)	(0.05)	(0.06)
Constant	0.37***	0.61***	0.28***	0.41***
	(0.05)	(0.05)	(0.04)	(0.06)
Observations	201	192	306	301

$^{\dagger}p<0.10$; $^{*}p<0.05$; $^{**}p<0.01$; $^{***}p<0.001$
OLS regression models.

Table 13: *YouGov 2017: Internationalists and Isolationists*

	Inter No Threat	Inter Threat	Iso No Threat	Iso Threat
Democratic Values	0.30***	0.14*	0.19***	0.21***
	(0.08)	(0.05)	(0.07)	(0.05)
Constant	0.29***	0.55***	0.26***	0.38***
	(0.05)	(0.04)	(0.05)	(0.04)
Observations	153	345	166	336

†p<0.10;*p<0.05; **p<0.01; ***p<0.001
OLS regression models.

Table 14: *Lucid 2019: Internationalists and Isolationists*

	Inter No Threat	Inter Threat	Iso No Threat	Iso Threat
Democratic Values	0.25***	0.05*	0.16**	0.13*
	(0.05)	(0.05)	(0.06)	(0.06)
Constant	0.56***	0.68***	0.34***	0.48***
	(0.04)	(0.03)	(0.04)	(0.04)
Observations	345	365	301	288

†p<0.10;*p<0.05; **p<0.01; ***p<0.001
OLS regression models.

Table 15: *Bilendi 2024: Internationalists and Isolationists*

	Inter No Threat	Inter Threat	Iso No Threat	Iso Threat
Democratic Values	0.15***	0.06**	0.18***	0.10**
	(0.03)	(0.03)	(0.03)	(0.03)
Constant	0.65***	0.73***	0.30***	0.44***
	(0.02)	(0.02)	(0.02)	(0.02)
Observations	868	896	1,106	1,087

†p<0.10;*p<0.05; **p<0.01; ***p<0.001
OLS regression models.

Table 16: *YouGov 2016: Interaction by Foreign Policy Orientation*

	Binary Inter	Binary Iso	Binary Inter	Binary Iso	Ordinal Inter	Ordinal Iso	Ordinal Inter	Ordinal Iso
Democratic Values	0.35***	0.26***	0.36***	0.25***	1.23***	0.97***	1.26***	0.96***
	(0.07)	(0.05)	(0.07)	(0.05)	(0.19)	(0.18)	(0.20)	(0.18)
Terrorism Threat	0.24***	0.13*	0.26***	0.10†	0.73**	0.40*	0.74**	0.32†
	(0.07)	(0.05)	(0.07)	(0.05)	(0.21)	(0.19)	(0.22)	(0.19)
Values x Threat	-0.21*	-0.00	-0.23*	0.02	-0.52†	-0.02	-0.55†	0.04
	(0.09)	(0.08)	(0.10)	(0.08)	(0.28)	(0.28)	(0.28)	(0.26)
Middle East Effect			-0.03	0.07			-0.16	0.22
			(0.06)	(0.05)			(0.17)	(0.17)
Africa Effect			-0.06	0.05			-0.17	0.27†
			(0.06)	(0.05)			(0.18)	(0.16)
Constant	0.37***	0.28***	0.25***	-0.06	4.02***	3.62***	3.70***	2.94***
	(0.05)	(0.04)	(0.08)	(0.07)	(0.15)	(0.13)	(0.25)	(0.25)
Observations	393	607	393	607	393	607	393	607
Controls			✓	✓			✓	✓

†p<0.10;*p<0.05; **p<0.01; ***p<0.001
OLS regression models. Controls for gender, college, partisanship, age, race, news interest.

Table 17: *YouGov 2017: Interaction by Foreign Policy Orientation*

	Binary Inter	Binary Iso	Binary Inter	Binary Iso	Ordinal Inter	Ordinal Iso	Ordinal Inter	Ordinal Iso
Democratic Values	0.29***	0.19*	0.28***	0.19*	0.94***	0.74**	0.91**	0.67*
	(0.08)	(0.07)	(0.08)	(0.08)	(0.26)	(0.27)	(0.27)	(0.28)
Terrorism Threat	0.26***	0.12*	0.22**	0.10†	0.92***	0.46*	0.85***	0.34
	(0.07)	(0.06)	(0.07)	(0.06)	(0.23)	(0.22)	(0.23)	(0.21)
Values x Threat	-0.16†	0.02	-0.14	0.04	-0.46	0.10	-0.44	0.25
	(0.09)	(0.09)	(0.09)	(0.09)	(0.30)	(0.33)	(0.31)	(0.33)
Middle East Effect			-0.01	-0.01			-0.01	0.04
			(0.05)	(0.05)			(0.16)	(0.19)
Africa Effect			-0.03	0.01			-0.08	0.09
			(0.05)	(0.05)			(0.17)	(0.19)
Constant	0.29***	0.26***	0.18*	0.09	3.81***	3.43***	3.61***	3.17***
	(0.05)	(0.09)	(0.08)	(0.07)	(0.20)	(0.17)	(0.27)	(0.26)
Observations	498	502	498	502	497	502	497	502
Controls			✓	✓			✓	✓

†p<0.10;*p<0.05; **p<0.01; ***p<0.001
OLS regression models. Controls for gender, college, partisanship, age, race, news interest.

Table 18: *Lucid 2019: Interaction by Foreign Policy Orientation*

	Binary Inter	Binary Iso	Binary Inter	Binary Iso	Ordinal Inter	Ordinal Iso	Ordinal Inter	Ordinal Iso
Democratic Values	0.25***	0.16**	0.24***	0.16**	0.98***	0.72***	0.93***	0.73***
	(0.05)	(0.06)	(0.05)	(0.06)	(0.16)	(0.19)	(0.16)	(0.19)
Terrorism Threat	0.12*	0.14*	0.11*	0.13*	0.47**	0.46*	0.43**	0.45*
	(0.05)	(0.06)	(0.05)	(0.08)	(0.17)	(0.19)	(0.16)	(0.19)
Values x Threat	-0.20**	-0.03	-0.19**	-0.03	-0.61**	-0.07	-0.55*	-0.09
	(0.07)	(0.08)	(0.07)	(0.08)	(0.22)	(0.26)	(0.22)	(0.26)
Constant	0.56***	0.34***	0.44***	0.09	4.59***	3.89***	4.32***	3.54***
	(0.04)	(0.04)	(0.06)	(0.06)	(0.12)	(0.26)	(0.18)	(0.21)
Observations	710	589	710	589	710	589	710	589
Controls			✓	✓			✓	✓

†p<0.10;*p<0.05; **p<0.01; ***p<0.001
OLS regression models. Controls for gender, college, partisanship, age, race, news interest.

Table 19: *Bilendi 2024: Interaction by Foreign Policy Orientation*

	Binary Inter	Binary Iso	Binary Inter	Binary Iso	Ordinal Inter	Ordinal Iso	Ordinal Inter	Ordinal Iso
Democratic Values	0.15***	0.18***	0.16***	0.17***	0.47***	0.63***	0.49***	0.62***
	(0.03)	(0.03)	(0.03)	(0.03)	(0.10)	(0.10)	(0.10)	(0.09)
Terrorism Threat	0.08*	0.13***	0.08*	0.14***	0.18†	0.47***	0.19†	0.48***
	(0.03)	(0.03)	(0.03)	(0.03)	(0.10)	(0.10)	(0.10)	(0.10)
Values x Threat	-0.09*	-0.08†	-0.10*	-0.07†	-0.13	-0.24†	-0.17	-0.24†
	(0.04)	(0.04)	(0.04)	(0.04)	(0.14)	(0.14)	(0.14)	(0.13)
Middle East Effect			-0.01	0.00			0.01	0.00
			(0.03)	(0.03)			(0.08)	(0.08)
Africa Effect			-0.02	-0.00			-0.06	-0.03
			(0.03)	(0.03)			(0.09)	(0.08)
Constant	0.65***	0.30***	0.55***	0.20***	5.00***	3.72***	4.66***	3.69***
	(0.02)	(0.02)	(0.04)	(0.04)	(0.08)	(0.14)	(0.13)	(0.11)
Observations	1,764	2,193	1,751	2,178	1,764	2,193	1,751	2,178
Controls			✓	✓			✓	✓

†p<0.10;*p<0.05; **p<0.01; ***p<0.001
OLS regression models. Controls for gender, college, partisanship, age, race, news interest.

Table 20: *Forced Choice Internationalist Measure (Lucid 2019 Only)*

	Inter No Threat	Iso No Threat	Inter Threat	Iso Threat	Inter Interaction	Iso Interaction
Democratic Values	0.26***	0.14*	0.10*	0.07	0.26***	0.14*
	(0.05)	(0.06)	(0.05)	(0.06)	(0.05)	(0.06)
Terrorism Threat					0.13**	0.14*
					(0.05)	(0.06)
Values x Threat					-0.16*	-0.07
					(0.07)	(0.09)
Constant	0.51***	0.38***	0.64***	0.51***	0.51***	0.38***
	(0.04)	(0.04)	(0.03)	(0.04)	(0.04)	(0.04)
Observations	385	261	392	261	777	522

†p<0.10;*p<0.05; **p<0.01; ***p<0.001
OLS regression models.

4.7 Disaggregated Regime Type and Human Rights Effects

In the fourth iteration of the experiment, respondents were randomized to read that the country's governance record involved one of the following: an authoritarian government with a reputation for violating human rights; an authoritarian government; a government with a reputation for violating human rights; a democratic government with a reputation for respecting human rights; a democratic government; or a government with a reputation for respecting human rights.

In Table 21, I compare these treatments to the "authoritarian regime with a reputation for violating human rights" as the reference category. By definition, all autocracies fail to respect key political rights of their citizens, since even by minimalist definitions of democracy a regime must guarantee these core rights to be classified as democratic. Furthermore, news coverage of US military aid to an authoritarian regime would almost always mention the regime type and rights abuses. As a result, the "authoritarian government with a reputation for violating human rights" is the most relevant comparison. Compared to this group, the results suggest that treatment effects are relatively similar when the democracy and human rights elements are either combined or separated. The separate and combined democracy and human rights treatments are all significantly distinguishable from the reference category; in addition, they are all distinguishable from the individual treatments of authoritarianism and violating human rights, and they are also both substantively and significantly different from the threat treatment.

Table 21: *Effects of Disaggregated Treatments are Relatively Similar (Bilendi 2024 Only)*

	Full Sample	No Threat	Terror Threat
Autocracy Only	0.06*	0.01	0.11**
	(0.03)	(0.04)	(0.04)
Violates HR Only	0.03	0.01	0.05
	(0.03)	(0.04)	(0.04)
Democracy Only	0.13***	0.14***	0.12***
	(0.03)	(0.04)	(0.04)
Respects HR Only	0.18***	0.19***	0.16***
	(0.03)	(0.04)	(0.04)
Democracy and Respects HR	0.18***	0.22***	0.14***
	(0.03)	(0.04)	(0.04)
Threat	0.07***		
	(0.02)		
Constant	0.45***	0.45***	0.52***
	(0.02)	(0.03)	(0.03)
Observations	3,958	1,975	1,983

†p<0.10;*p<0.05; **p<0.01; ***p<0.001
OLS regression models.

However, two additional patterns are worth commenting on. First, the individual

human rights treatment produces a larger effect than the individual democracy treatment, especially when comparing respect for human rights to violations of human rights and democracy to autocracy. The effect of the individual human rights treatment is also comparable in size to the combined democracy and human rights treatment. Second, the individual democracy treatment also appears to be less robust than the individual human rights treatment to the terrorism threat, especially when comparing them to the individual rights violation treatment and the individual autocracy treatment.

As a result, it may be the case that mentioning violations of human rights will have more of an impact on Americans' attitudes than mentioning authoritarianism. I discuss this possibility in the main paper. One substantive implication of this pattern is that democracies with problematic human rights records – e.g., Iraq or Israel – will likely be viewed less favorably by the American public as candidates for military aid.

However, this possibility does not detract from the primary conclusions of the paper about the effects of alignment with democratic values on attitudes toward military aid. Since all autocracies have reputations for violating human rights, and since democracies in general are better at protecting rights, the combined treatments remain useful in their own right. Furthermore, the consistently lower support of the "authoritarian regime with a reputation for violating human rights" suggests that the American public will be particularly reluctant to send military aid to abusive authoritarian regimes like those in Egypt or Saudi Arabia, and that this reluctance will be robust to the presence of terrorism threats.

4.8 Effects with Militant and Cooperative Internationalists

I code respondents as militant or cooperative internationalists based on their responses to two separate questions on the 2024 Bilendi survey. The question to gauge militant tendencies asks about war as a solution to international problems, and the question to gauge cooperative tendencies asks about the importance of cooperating with other nations on issues such as overpopulation, hunger, and pollution. The question wording was as follows: *Militant:* "Going to war is unfortunate but sometimes the only solution to international problems." *Cooperative:* "It is essential for the United States to work with other nations to solve problems such as over-population, hunger, and pollution."

I use these questions to create two measures of militant and cooperative internationalists: the first codes respondents as militant and/or cooperative if they proactively agree with the respective statements, and the second one also requires them to answer the standalone internationalist question affirmatively. Results are reported in Tables 22 and 23, with the first measure in the first four columns and the second measure in the second four columns.

Table 22: *Effects for **Cooperative** Internationalists (Bilendi 2024 Only)*

	Full Sample (1)	No Threat (1)	Terror Threat (1)	Interaction Model (1)	Full Sample (2)	No Threat (2)	Terror Threat (2)	Interaction Model (2)
Democratic Values	0.15***	0.20***	0.09***	0.20***	0.11***	0.18***	0.05	0.18***
	(0.02)	(0.03)	(0.02)	(0.03)	(0.02)	(0.03)	(0.03)	(0.03)
Terrorism Threat	0.08***			0.13**	0.04†			0.11***
	(0.02)			(0.03)	(0.02)			(0.03)
Values x Threat				-0.11**				-0.13**
				(0.04)				(0.04)
Constant	0.54***	0.52***	0.65***	0.51***	0.71***	0.67***	0.78***	0.67***
	(0.02)	(0.02)	(0.02)	(0.02)	(0.02)	(0.03)	(0.02)	(0.03)
Observations	2,769	1,391	1,378	2,769	1,433	710	723	1,433

†p<0.10;*p<0.05; **p<0.01; ***p<0.001; OLS regression models.

Table 23: *Effects for **Militant** Internationalists (Bilendi 2024 Only)*

	Full Sample (1)	No Threat (1)	Terror Threat (1)	Interaction Model (1)	Full Sample (2)	No Threat (2)	Terror Threat (2)	Interaction Model (2)
Democratic Values	0.12***	0.16***	0.09***	0.16***	0.08***	0.11**	0.06†	0.11**
	(0.02)	(0.03)	(0.03)	(0.03)	(0.02)	(0.04)	(0.03)	(0.04)
Terrorism Threat	0.07*			0.10***	0.02			0.04
	(0.02)			(0.03)	(0.02)			(0.04)
Values x Threat				-0.07†				-0.05
				(0.04)				(0.05)
Constant	0.58***	0.56***	0.66***	0.56***	0.75***	0.74***	0.78***	0.74***
	(0.02)	(0.02)	(0.02)	(0.02)	(0.02)	(0.03)	(0.03)	(0.03)
Observations	1,925	936	989	1,925	1,040	499	541	1,040

†p<0.10;*p<0.05; **p<0.01; ***p<0.001; OLS regression models.

Overall, the results suggest that more cooperative or militant internationalists react similarly to the treatments.

4.9 Descriptive Characteristics of Internationalists and Isolationists by Survey

Table 24: *Internationalist Features by Survey*

	YouGov 2016	YouGov 2017	Lucid 2019	Bilendi 2024
Republican	29%	25%	29%	32%
Democratic	48%	35%	43%	45%
Independent	23%	29%	23%	23%
University Educated	41%	38%	42%	47%
Male	43%	45%	48%	54%

Table 25: *Isolationist Features by Survey*

	YouGov 2016	YouGov 2017	Lucid 2019	Bilendi 2024
Republican	28%	20%	29%	32%
Democratic	31%	36%	30%	33%
Independent	41%	32%	31%	35%
University Educated	39%	35%	38%	48%
Male	46%	45%	48%	47%

4.10 Effects by Foreign Policy Orientation and Age

The table shows the interaction between the democratic values and threat treatments for internationalists and isolationists, first among respondents aged 18 to 30 and then among respondents aged 31 and older. The two age groups do differ, with older respondents generally more responsive to the treatments and more affected by their interaction. However, the differences between internationalists and isolationists in the two age groups are very similar, insofar as the interaction is substantively similar by foreign policy orientation for both age groups. This result suggests that the muted difference between internationalists and isolationists on the fourth survey is not driven by the oversampling of younger respondents relative to the other three implementations of the experiment.

Table 26: *Democratic Values and Threat Interaction by Age Group*

	Age 18-30 Internationalist	Age 18-30 Isolationist	Age 31+ Internationalist	Age 31+ Isolationist
Democratic Values	0.06	0.12**	0.26***	0.23***
	(0.04)	(0.04)	(0.04)	(0.04)
Terrorism Threat	0.03	0.06	0.13**	0.19***
	(0.04)	(0.04)	(0.05)	(0.04)
Values x Threat	-0.05	-0.02	-0.14*	-0.13*
	(0.06)	(0.06)	(0.06)	(0.06)
Constant	0.72***	0.34***	0.59***	0.28***
	(0.03)	(0.03)	(0.03)	(0.03)
Observations	932	957	832	1,236

$^{\dagger}p<0.10$; $^{*}p<0.05$; $^{**}p<0.01$; $^{***}p<0.001$; OLS regression models.

4.11 Democratic Values, Threat, and Education

On the Bilendi survey, there is more polarization by educational attainment in terms of reactions to the democratic values and threat treatments and in terms of the interaction between these treatments. This may reflect broader political polarization among educational groups in US politics and could account for the different internationalist / isolationist results in the fourth iteration of the experiment.

Table 27: *Democratic Values and Threat Interaction by Educational Attainment*

	Bilendi Survey University	Bilendi Survey No University	Other Surveys University	Other Surveys No University
Democratic Values	0.22***	0.12***	0.26***	0.24***
	(0.03)	(0.03)	(0.04)	(0.03)
Terrorism Threat	0.17***	0.06*	0.20***	0.13***
	(0.03)	(0.03)	(0.04)	(0.03)
Values x Threat	-0.14***	-0.04	-0.08	-0.11*
	(0.04)	(0.04)	(0.05)	(0.04)
Internationalist	0.29***	0.32***	0.20***	0.13***
	(0.02)	(0.02)	(0.03)	(0.02)
Constant	0.30***	0.34***	0.28***	0.29***
	(0.02)	(0.02)	(0.03)	(0.03)
Observations	1,879	2,078	1,287	2,012

†p<0.10;*p<0.05; **p<0.01; ***p<0.001; OLS regression models.

5 Descriptive Results

5.1 Support for Military Aid

Table 28: *Correlates of Support for Military Aid*

	Lucid 2019	Lucid 2021	Lucid 2023	Bilendi 2024
Internationalist	0.38***	0.40***	0.32***	0.32***
	(0.03)	(0.02)	(0.03)	(0.02)
Republican	0.13***	0.09***	0.05†	0.05*
	(0.03)	(0.03)	(0.03)	(0.02)
Democrat	0.14***	0.04	0.08**	0.09***
	(0.03)	(0.03)	(0.03)	(0.02)
Democracy Important	0.00	0.04†	0.01	0.06***
	(0.03)	(0.02)	(0.03)	(0.02)
Male	0.06*	0.10***	0.05*	0.07***
	(0.03)	(0.02)	(0.02)	(0.02)
Above Median Age	0.03	-0.01	-0.09***	0.02
	(0.03)	(0.02)	(0.03)	(0.02)
University	0.01	0.02	0.00	0.03^{dagger}
	(0.03)	(0.02)	(0.03)	(0.02)
White	-0.06*	0.00	0.02	-0.01
	(0.03)	(0.02)	(0.03)	(0.02)
News Interest	0.08**	0.08***	0.15***	0.06***
	(0.03)	(0.02)	(0.03)	(0.02)
Constant	0.23***	0.20***	0.24***	0.26***
	(0.04)	(0.03)	(0.03)	(0.02)
Observations	1,299	1,983	1,532	3,911

†p<0.10;*p<0.05; **p<0.01; ***p<0.001
OLS regression models.

5.2 Support for Democracy Promotion

Table 29: *Correlates of Support for Democracy Promotion*

	Lucid 2019	Lucid 2021	Lucid 2023	Bilendi 2024
Internationalist	0.31***	0.33***	0.23***	0.21***
	(0.03)	(0.02)	(0.02)	(0.01)
Republican	-0.01	0.03	0.04	0.04*
	(0.03)	(0.03)	(0.03)	(0.02)
Democrat	0.13***	0.06*	0.12***	0.15***
	(0.03)	(0.02)	(0.03)	(0.02)
Democracy Important	0.19***	0.15***	0.13***	0.16***
	(0.03)	(0.02)	(0.02)	(0.01)
Male	0.07**	0.07***	0.03	0.05***
	(0.02)	(0.02)	(0.02)	(0.01)
Above Median Age	0.01	0.07***	-0.00	0.06***
	(0.03)	(0.02)	(0.02)	(0.02)
University	0.04†	0.05**	0.08***	0.06***
	(0.02)	(0.02)	(0.02)	(0.01)
White	-0.00	0.03	0.01	0.04*
	(0.03)	(0.02)	(0.02)	(0.02)
News Interest	0.12***	0.10***	0.18***	0.08***
	(0.03)	(0.02)	(0.03)	(0.02)
Constant	0.22***	0.22***	0.31***	0.28***
	(0.03)	(0.03)	(0.03)	(0.02)
Observations	1,299	1,984	1,532	3,911

†p<0.10; *p<0.05; **p<0.01; ***p<0.001
OLS regression models.

5.3 Support for Working with Autocrats against Terrorism

Table 30: *Correlates of Support for Working With Autocrats*

	Lucid 2019	Lucid 2021	Lucid 2023	Bilendi 2024
Internationalist	0.21***	0.28***	0.15***	0.19***
	(0.02)	(0.02)	(0.02)	(0.01)
Republican	0.07**	0.06*	0.09***	0.09***
	(0.03)	(0.03)	(0.03)	(0.02)
Democrat	0.04	0.05*	0.06*	0.09***
	(0.03)	(0.02)	(0.03)	(0.02)
Democracy Important	0.07***	0.09***	0.00	0.04*
	(0.02)	(0.02)	(0.02)	(0.02)
Male	0.04†	0.05*	0.04†	0.07***
	(0.02)	(0.02)	(0.02)	(0.02)
Above Median Age	0.05*	0.12***	0.03	0.11***
	(0.02)	(0.02)	(0.02)	(0.02)
University	0.00	0.01	0.05*	0.01
	(0.02)	(0.02)	(0.02)	(0.01)
White	-0.00	0.05*	0.01	0.01
	(0.03)	(0.02)	(0.03)	(0.02)
News Interest	0.01	0.01	0.14***	0.04**
	(0.02)	(0.02)	(0.03)	(0.02)
Constant	0.56***	0.37***	0.47***	0.41***
	(0.03)	(0.03)	(0.03)	(0.02)
Observations	1,299	1,981	1,532	3,911

†p<0.10; *p<0.05; **p<0.01; ***p<0.001
OLS regression models.

5.4 Descriptive Analysis of Militant and Cooperative Internationalists

The descriptive results show that internationalists are more likely than isolationists to support military aid; in addition, they are also more likely to prefer that US foreign policy promote democracy and that the United States work with both autocracies and democracies to manage terrorism. Are these attitudes more pronounced among certain types of internationalists? As discussed in the main text, prior research establishes that some internationalists prefer a more militant approach to international politics, whereas others prioritize cooperative engagement (Chanley 1999). These militant and cooperative internationalists often differ on their preferences toward foreign policy tools and decisions (Holsti and Rosenau 1990). However, using the aforementioned two questions on the 2024 Bilendi survey that gauged militant and cooperative tendencies, I find little difference in attitudes toward military aid, democracy promotion, and counterterrorism alliances with autocracies.

Table 31: *Cooperative and Militant Internationalists Align on Military Aid*

	Aid Foreign Militaries (1)	Promote Democracy (1)	Ally with Dem and Autoc (1)	Aid Foreign Militaries (2)	Promote Democracy (2)	Ally with Dem and Autoc (2)
Cooperative	0.24***	0.20***	0.25***	0.21***	0.15***	0.19***
	(0.02)	(0.02)	(0.02)	(0.02)	(0.02)	(0.02)
Militant	0.18***	0.17***	0.14***	0.20***	0.14***	0.06***
	(0.02)	(0.01)	(0.01)	(0.02)	(0.02)	(0.02)
Constant	0.19***	0.20***	0.30***	0.30***	0.30***	0.42***
	(0.02)	(0.02)	(0.02)	(0.02)	(0.02)	(0.02)
Controls	✓	✓	✓	✓	✓	✓
Observations	3,910	3,910	3,910	3,910	3,910	3,910

†p<0.10;*p<0.05; **p<0.01; ***p<0.001
OLS regression models.
Controls for Republican, Democrat, importance of democracy, gender, above median age, university education, ethnicity, and news interest.

As described in the experimental section above, the question to gauge militant tendencies asks about war as a solution to international problems, and the question to gauge cooperative tendencies asks about the importance of cooperating with other nations on issues such as overpopulation, hunger, and pollution. The question wording was as follows: *Militant:* "Going to war is unfortunate but sometimes the only solution to international problems." *Cooperative:* "It is essential for the United States to work with other nations to solve problems such as over-population, hunger, and pollution."

For the analysis, I create binary variables for cooperation and militancy based solely on supportive answers to each question, and then based on answers to each question combined with responses to the standalone internationalism question. I then regress the three outcomes on these measures. Results are reported in Table 31. They show that both militant and cooperative tendencies are associated with similar increases in support for aiding foreign militaries, promoting democracy, and aligning with autocracies to address terrorism

using both constructions of the variables. The one exception is the substantively smaller relationship between militant internationalists and working with autocracies and democracies to fight terrorism when using the second measure of militant internationalism.

Overall, these results suggest that even if militant and cooperative internationalists approve of these statements for different reasons, they still arrive at similar levels of support relative to isolationists. Military aid involves hard power but also cooperation with other countries, which may make it an appealing tool to both groups. Likewise, militant and cooperative internationalists may both view democracy promotion as an important US objective, even if they differ over how to attain it. And if both groups perceive counterterrorism as an important US interest, they may want the US government to work with authoritarian governments to address it, even if they do not fully agree on what those relationships would entail. In other words, based on these results, it seems likely that internationalists with both militant and cooperative tendencies will be relatively prone to conflicted views about the relevance of democratic values to military aid when faced with threats to global security.
