# Supplementary figures and images for: Who Gets the Guns? How Democratic Values and Security Threats Affect American Attitudes Toward Military Aid
Source: J Conflict Resolut. 2025 Oct 28;70(6):986–1017. doi: 10.1177/00220027251388634 (PMC13211145; doi:10.1177/00220027251388634)

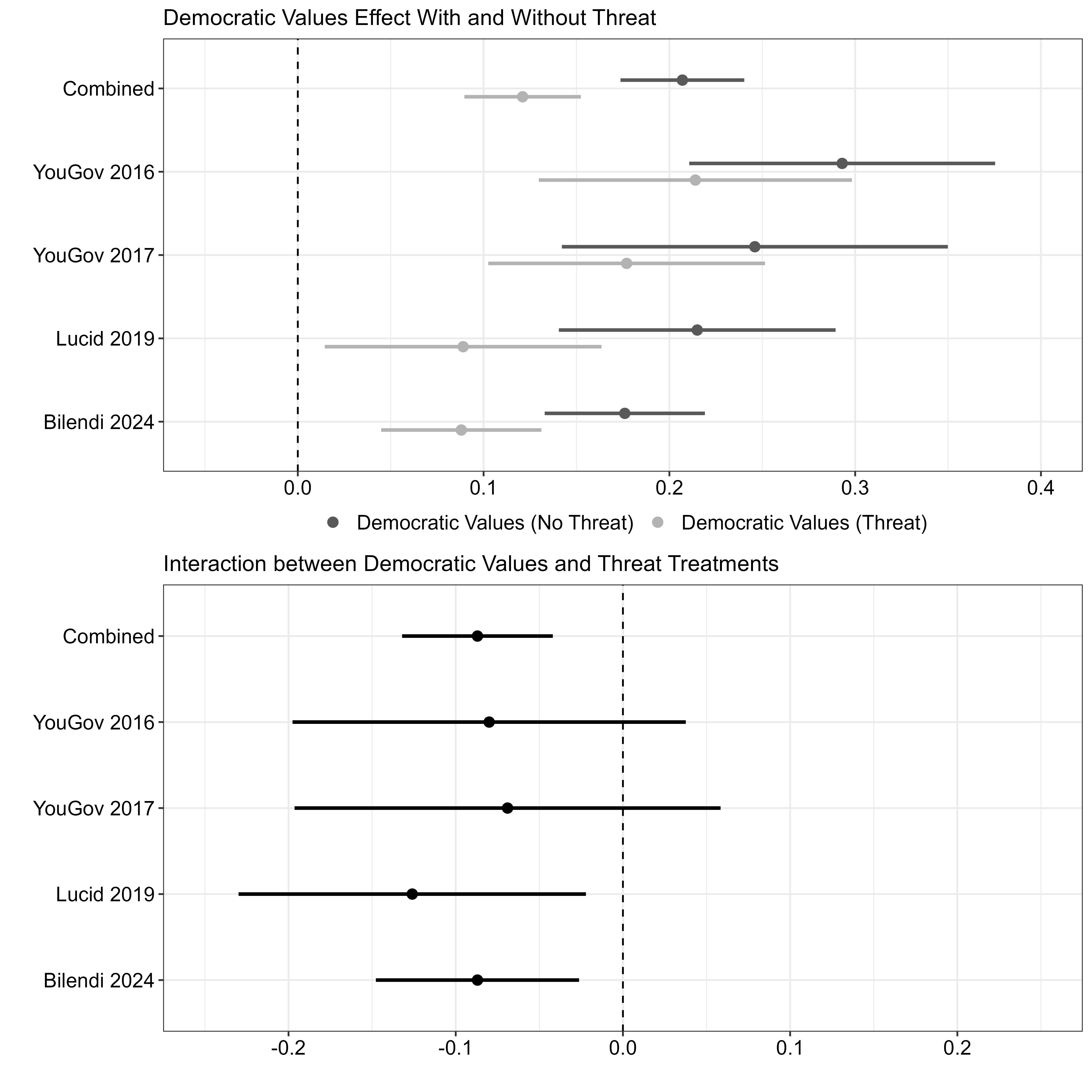

Supplement: Supplemental Material - Who Gets the Guns? How Democratic Values and Security Threats Affect American Attitudes Toward Military Aid [file sj-zip-2-jcr-10.1177_00220027251388634.zip › conditional_interaction_plots.jpg]

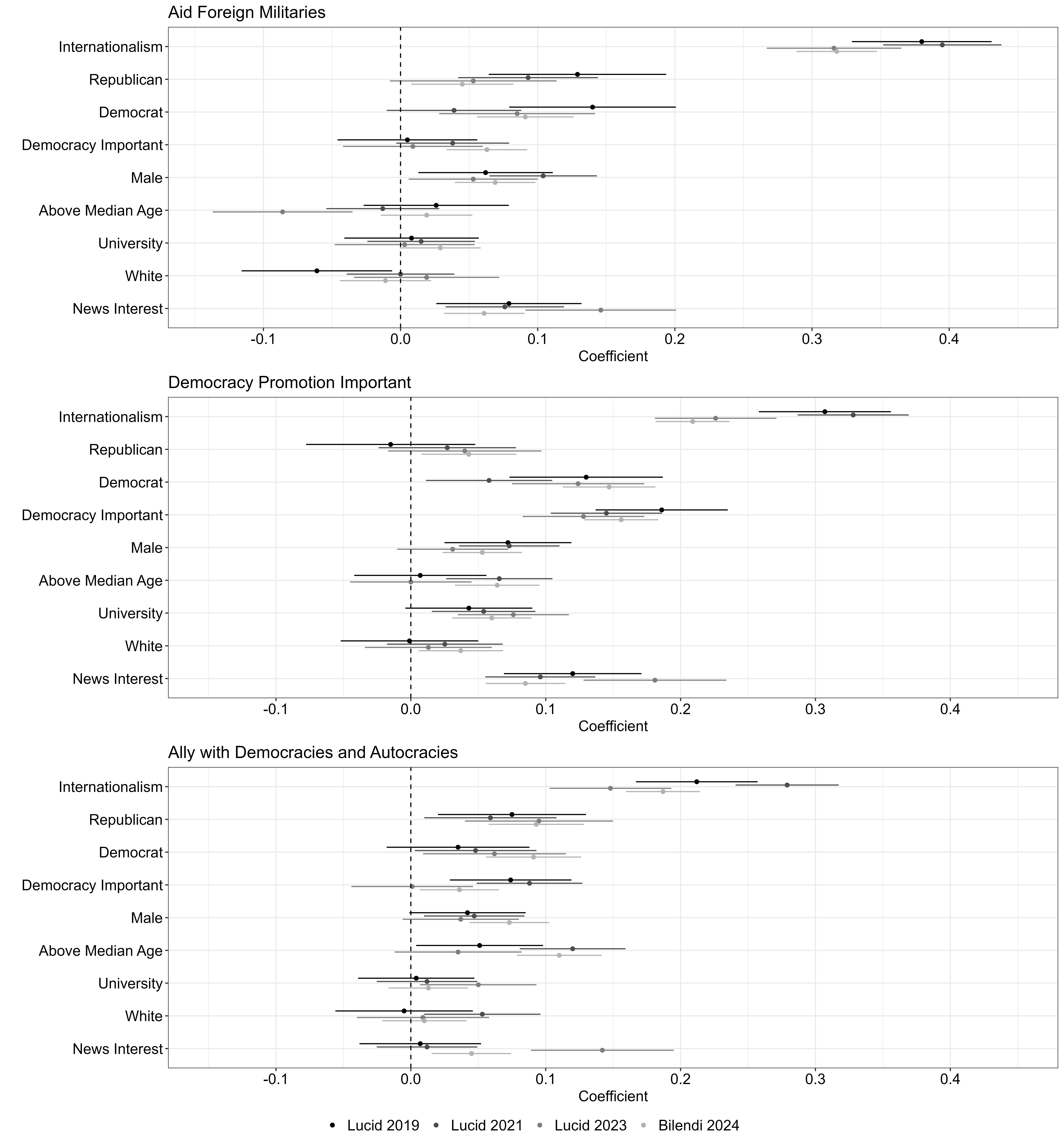

Supplement: Supplemental Material - Who Gets the Guns? How Democratic Values and Security Threats Affect American Attitudes Toward Military Aid [file sj-zip-2-jcr-10.1177_00220027251388634.zip › descriptive_military_aid_plots.jpg]

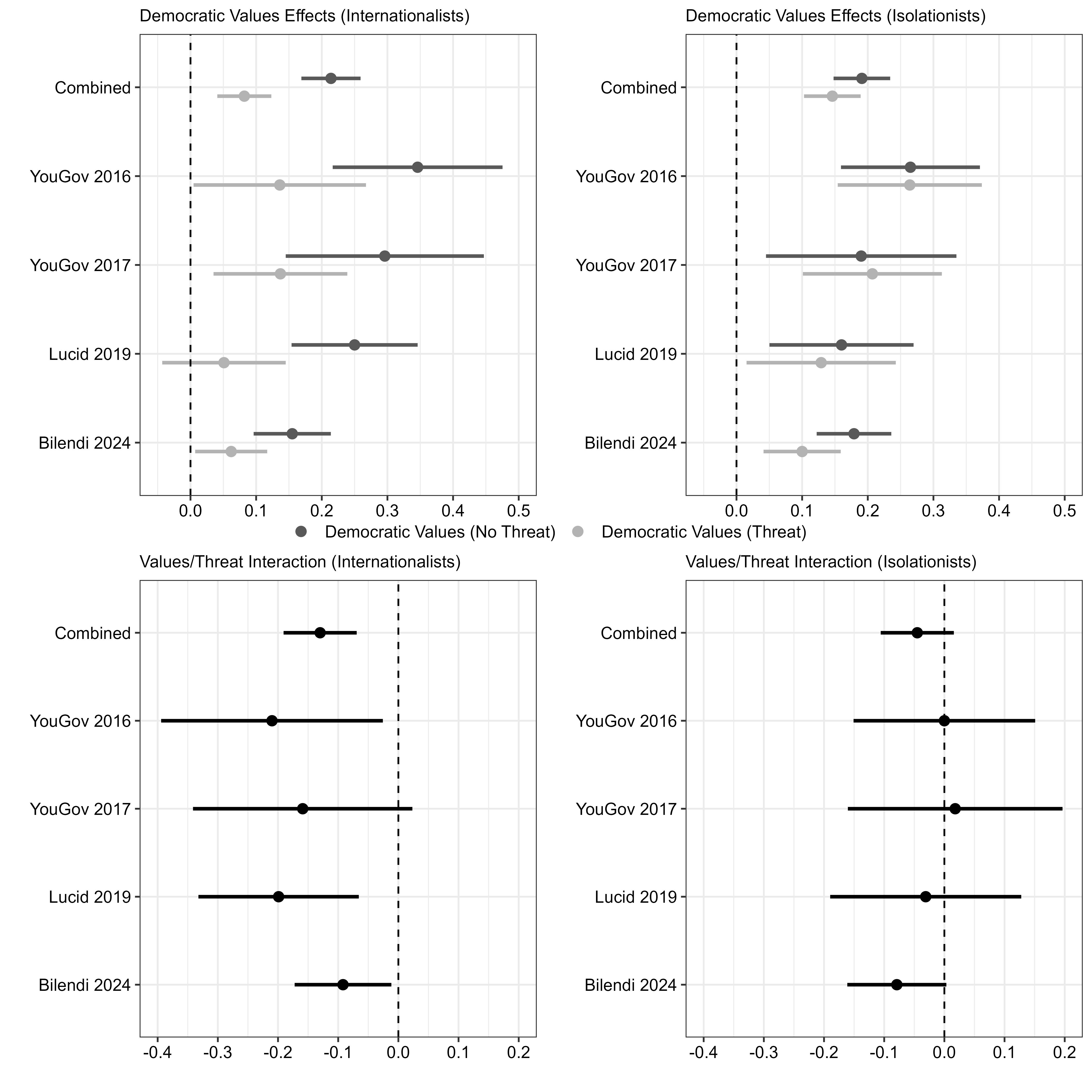

Supplement: Supplemental Material - Who Gets the Guns? How Democratic Values and Security Threats Affect American Attitudes Toward Military Aid [file sj-zip-2-jcr-10.1177_00220027251388634.zip › inter_iso_combined_plots.jpg]

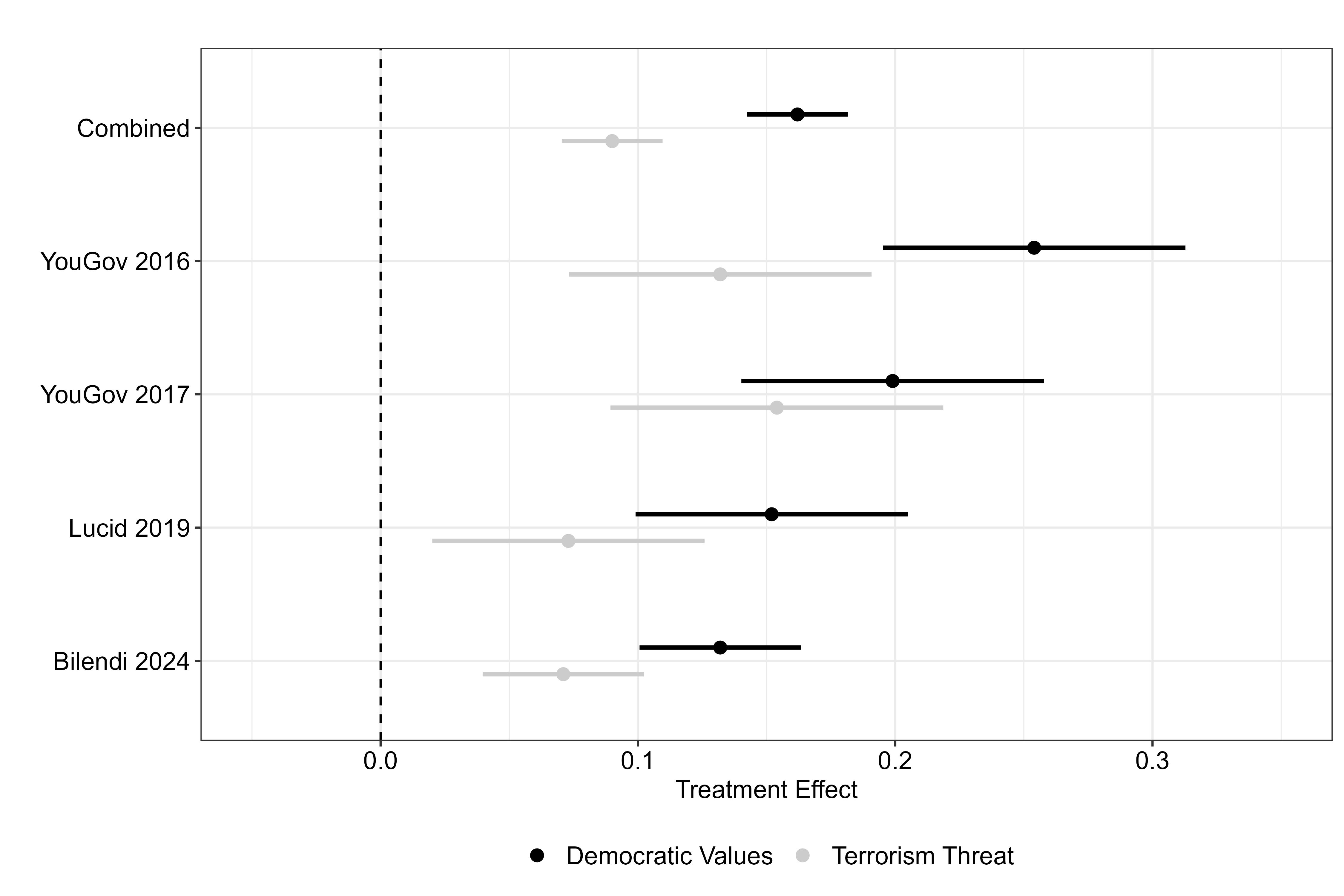

Supplement: Supplemental Material - Who Gets the Guns? How Democratic Values and Security Threats Affect American Attitudes Toward Military Aid [file sj-zip-2-jcr-10.1177_00220027251388634.zip › main_effect_plot.jpg]
